# Diversity in the Globally Distributed Diatom Genus *Chaetoceros* (Bacillariophyceae): Three New Species from Warm-Temperate Waters

**DOI:** 10.1371/journal.pone.0168887

**Published:** 2017-01-13

**Authors:** Yang Li, Atchaneey Boonprakob, Chetan C. Gaonkar, Wiebe H. C. F. Kooistra, Carina B. Lange, David Hernández-Becerril, Zuoyi Chen, Øjvind Moestrup, Nina Lundholm

**Affiliations:** 1 Guangzhou Key Laboratory of Subtropical Biodiversity and Biomonitoring, College of Life Science, South China Normal University, Guangzhou, P. R. China; 2 Natural History Museum of Denmark, University of Copenhagen, Copenhagen, Denmark; 3 Department of Biology, Faculty of Science, Ramkhamhaeng University, Bangkok, Thailand; 4 Integrative Marine Ecology, Stazione Zoologica Anton Dohrn, Naples, Italy; 5 Department of Oceanography and Center COPAS Sur-Austral, University of Concepción, Concepción, Chile; 6 Instituto de Ciencias del Mar y Limnologia, Universidad Nacional Autónoma de México, Cd. Universitaria, Coyoacán, Cd. de México, México; 7 Section of Marine Biology, Institute of Biology, University of Copenhagen, Copenhagen, Denmark; University of Cambridge, UNITED KINGDOM

## Abstract

*Chaetoceros* is one of the most species rich, widespread and abundant diatom genera in marine and brackish habitats worldwide. It therefore forms an excellent model for in-depth biodiversity studies, assessing morphological and genetic differentiation among groups of strains. The global *Chaetoceros lorenzianus* complex presently comprises three species known to science. However, our recent studies have shown that the group includes several previously unknown species. In this article, 50 strains, mainly from high latitudes and from warm-temperate waters, were examined morphologically and genetically and the results compared with those of field studies from elsewhere. The strains clustered into five groups, two of which are formed by *C*. *decipiens* Cleve and *C*. *mitra* (Bailey) Cleve, respectively. Their species descriptions are emended based on samples collected close to the type localities. The three other groups are formed by new species, *C*. *elegans* sp. nov., *C*. *laevisporus* sp. nov. and *C*. *mannaii* sp. nov. Characters used to distinguish each species are: orientation of setae, shape and size of the apertures, shape, size and density of the poroids on the setae and, at least in some species, characters of the resting spores. Our aim is to cover the global species diversity in this complex, as correct species delineation is the basis for exploring biodiversity, distribution of organisms, interactions in the food web and effects of environmental changes.

## Introduction

Diatoms constitute one of the most abundant and diverse phytoplankton groups, with estimates of 200.000 species [1]. These numbers are rough estimates, and the present number of species described is only 12.000 [2]. Recent studies on marine genera such as *Pseudo-nitzschia* and *Skeletonema* also show that species diversity is much higher than previously known e.g. [3, 4]. Is this a general trend that applies to other marine diatoms? To answer this question, we chose *Chaetoceros* as model for a taxonomic study on species diversity.

*Chaetoceros* is one of the largest genera of diatoms in the marine phytoplankton, and its many species are widely distributed, some even cosmopolitan. Species have been described since 1844, and the *Chaetoceros* taxa in Algaebase presently number 529 ([5] accessed Apr. 2016).

Members of the genus are usually easily recognized to genus level by the cells forming chains in which cells are separated by apertures, and by long setae protruding from each of the four corners of the cells. Species with solitary cells are also known. Species identification, however, can be difficult. The majority of taxa have, as most other diatom species, been described from light microscopy only. We have for some time been engaged in combining morphological and molecular data of *Chaetoceros* species, with the aim of obtaining a better idea of the diversity.

The genus *Chaetoceros* is often divided into subgenera and sections, based on morphological characters. While the validity of some of these subgroups is convincing also in a phylogenetic context, others are less so. Molecular data in a phylogenetic context is needed to further refine the number and circumscription of subgroups, and to obtain an idea of their phylogenetic relation to one another.

In the present article we report on species referred to section *Dicladia* (Ehrenberg) Gran, a small section of only three species, all marine: *C*. *lorenzianus*, *C*. *decipiens* and *C*. *mitra*. Cells are characterized by forming straight chains with stiff setae; they each contain typically 4–10 chloroplasts, and the setae on the terminal cells of chains are typically coarser than those on the intercalary cells, and oriented differently [6]. The name *Dicladia* (“two branches”) was coined by Ehrenberg for what we now know are the two conspicuous ornaments on resting spores of some of the species in the group.

The three known species were described within the short span of 17 years, 1856–1873, and no further species have been added to the section since then. Our new studies, which began some 135 years later, have shown, however, that the number of species in the section is greatly underestimated. In the present article we report on three new species discovered in warm-temperate waters.

Of the three known species in the section, *Chaetoceros decipiens* was described from the North Atlantic and the Davis Strait by Cleve [7], and *C*. *lorenzianus* from the Adriatic Sea and the Indian Ocean by Grunow [8]. Both species have subsequently been reported from geographically widely separated localities, from polar areas to warm waters, sometimes even occurring together, e.g. in Danish coastal waters [9], Narragansett Bay of Rhode Island [10], St Lawrence Estuary [11], Gulf of California [12], Sea of Japan [13], Chinese coastal waters [14], Southern Gulf of Mexico [15] and Guangdong coastal waters [16]. Hasle & Syvertsen [6] described *C*. *decipiens* as cosmopolitan and *C*. *lorenzianus* as a warm water species.

The third species, *Chaetoceros mitra*, was first described from the valve of a resting spore collected in the Sea of Kamtschatka by Bailey ([17] as *Dicladia mitra*) and it is less commonly reported: Greenland ([18], present study) and Norway ([19], present study). It is believed to be a northern cold-water species [6].

The morphological differences between the three species are small, and the characters used for species delineation have varied over time and among authors. Identification can therefore be challenging [10, 12, 20, 21]. Strains obtained from a series of localities (South China Sea, East China Sea, Yellow Sea, Thailand, Mexico, Chile) could not with certainty be allocated to any of the three described species, and material agreeing perfectly with the description of *C*. *lorenzianus* has never been characterized in detail morphologically or genetically. A detailed morphological and molecular investigation based on monoclonal cultures is therefore required.

In the present study, monoclonal cultures were established from samples collected in localities in tropical, temperate and polar areas, those from polar areas near the type locality of *C*. *decipiens* and close to the locality where vegetative cells of *C*. *mitra* were first observed. Light- and electron microscopy as well as phylogenetic analyses were conducted and the type material of both *C*. *lorenzianus* and *C*. *decipiens* was examined.

## Materials and Methods

### Cultures and other material

Live samples were collected from localities in Thailand, China, Mexico, Chile, Italy, Norway, Denmark, the Norwegian Sea, the Denmark Strait and Greenland (S1 Table). In Mexico, the Instituto de Ciencias del Mar y Limnología and Universidad Nacional Autónoma de México have a general sampling permission. In Thailand, sampling was permitted via the University of the Royal Thai Government. In Italy, cells were collected at the LTER Marechiara operated by the Stazione Zoologica in the Gulf of Naples, and no specific permission is required as SZN has the right to sample there. For the Chilean sample sites near Las Cruces, ECIM, Las Cruces gave the right to sample on the permission given to them by the Pontifical University of Santiago de Chile, which has the authority to give such permissions. The Chilean sample from off Concepcion comes from the site of the Oceanographic Time Series of the University of Concepción. No specific permission is required as the University of Concepcion has the right to sample there. Permission to sample in Greenland was given by Departementet for Erhverv og Arbejdsmarked, Government of Greenland which issues sampling permission for all Greenlandic waters. The sampling was in accordance with Norwegian laws. No specific permissions are required to sample in Chinese coastal regions or Danish waters when the species or the area is not protected.

Using a glass micro-pipette, single cells or chains of *Chaetoceros* were isolated from plankton net samples (mesh size 20 μm) and water samples, using an inverted microscope (Nikon TMS, Tokyo, Japan). The cultures were maintained in L1 or f/2-medium at a salinity of 30–34 [22]. Monoclonal cultures were incubated in a 16:8 light:dark (L:D) cycle, the illumination provided by cool fluorescent lamps or incubated in a north-facing window. Cultures were incubated at 20–26°C, 15 ± 1°C or 4 ± 1°C, depending on their origin. Induction of resting spore formation was attempted at least three times in several strains (if available) of each species by inoculation of cells into L1 or f/2 medium prepared without nitrogen. For DNA sequencing, culture aliquots were concentrated and frozen or processed directly.

Type material of *C*. *lorenzianus* was acquired from the Grunow collection, Naturhistorisches Museum, Vienna, with kind help from Anton Igersheim. In Grunow’s accession book under number 501 (Grunow 501) the locality of *C*. *lorenzianus*: “Porto piccolo bei Castel Muschio/1^st^ January” is mentioned beside a slide in a capsule (Acqu 1901/3674) with a coverslip of mica. The capsule is glued on a small paper sheet and next to the capsule is a sketch of *C*. *lorenzianus* made by Grunow. The sketch is very similar to fig 13, plate 14(V) [8] and probably a draft for the drawing. In Grunow’s “Bildersammlung” (collection of images) a similar sketch (Grunow 501) is found. Raw material of Grunow 501 is not available (A. Igersheim pers. comm.), and thus electron microscopy of the material was not possible. The slide with mica was borrowed from the Grunow collection and observed under the light microscope.

Material from the P.T. Cleve’s collection, Stockholm University, Sweden, related to *C*. *decipiens* based on locality, was kindly provided by Marianne Hamnede, who informed us that no type has been selected for *C*. *decipiens*. The material was from the North Atlantic Ocean and the Davis Strait, collected by Th. M. Fries and comprised permanent slides in which we observed *C*. *decipiens*. The raw material acquired differed completely from the material on the slides and no *Chaetoceros* was observed, thus only light microscopy was possible to perform on the material.

### Morphological observations

Light microscopical observations were done using a Zeiss Axioplan light microscope (Zeiss, Oberkochen, Germany) equipped with Nomarski interference contrast and an AxioCam HRc digital camera, and an Olympus BX53 (Olympus, Tokyo, Japan) with an Olympus DP27 camera. The pervalvar axis and the length of the aperture were measured in LM micrographs on chains in broad girdle view, in the middle of the apical axis.

For transmission electron microscopy, concentrated live material was rinsed three times in distilled water, with centrifugation at 2000 rpm for 10 min or 5000 rpm for 20 min between each change. The material was then either 1) dried onto Formvar-coated copper grids or 2) subjected to acid treatment. The ratio of sample to acids was 1:1:2 (Sample: HNO_3_:H_2_SO_4_). This mixture was boiled for a few seconds and washed again with distilled water until the pH of the material was neutralised. The material was dried onto Formvar-coated copper grids and used for examination in a JEOL-1010 transmission electron microscope (TEM) (Jeol, Tokyo, Japan), or a TEM LEO 912AB (LEO, Oberkochen, Germany).

For scanning electron microscopy, the rinsed material was filtered onto IsoporeTM membrane filters, pore size 8 μm (Merck Millipore, Billerica, Massachusetts, USA) and dehydrated in an ethanol series, 10 min in each change of 30, 50, 70, 96% ethanol, followed by 15 min in 99.9% ethanol and 30 min in absolute ethanol, before critical point drying in a BAL-TEC CPD 030 critical point drier (Balzer, Liechtenstein). The filters were attached to stubs with double-sticky carbon tape (12 mm diam., Agar Scientific, Northriding, England) and sputter coated for 100 s with gold-palladium in a JEOL JFC-2300HR coating unit (Jeol, Tokyo, Japan) before examination in a JEOL JSM-6335F scanning electron microscope (SEM) (Jeol, Tokyo, Japan) or a JEOL JSM-6500F (JEOL-USA Inc., Peabody, MA, USA).

For statistical analyses of the morphometrics, one-way ANOVA with Bonferroni-Holm post hoc tests were done using Daniel’s XL Toolbox add-in for Excel, version 6.22 [23].

Terminology was based on [10, 24].

### Phylogenetic analyses

DNA extraction was performed as described in [25], and the hypervariable D1–D3 region of the nuclear large subunit ribosomal RNA-encoding gene region (LSU rRNA gene) was amplified and sequenced using the primers D1R-F [26] and D3B-R′ [27]. PCR conditions included 35 cycles, each comprising 94°C for 35 s, 58°C for 35 s and 72°C for 50 s. The relatively conservative region SSU rRNA was amplified using the primers SSU-F and SSU-R [28] and 38 cycles, each comprising 94°C for 20 s, 54°C for 30 s and 72°C for 2 min. PCR products were purified using QIAquick PCR Purification Kit (Qiagen, Germany) as recommended by the manufacturer and analyzed on an AB3130xl automated sequencer (Applied Biosystems) or sent to Macrogen (http://dna.macrogen.com). For SSU sequencing, besides the same primers as for PCR, SSU515+, SSU1004+, SSU1451+, SSU1147- and SSU568- were used alternatively [29].

Sequences were aligned together with similar sequences from Genbank (for accession numbers, see S1 Table) and edited manually in BioEdit [30], and *C*. *diadema* was chosen as outgroup taxon based on analyses of a large *Chaetoceros* alignment (not shown). All base pair positions were included in the analyses. The analyses, except Bayesian analyses (MrB), were performed using PAUP* version 4.0b.8 [31]. Maximum parsimony (MP) analyses were done using heuristic searches with random addition of sequences (100 replicates) and a branch-swapping algorithm (TBR, Tree Bisection Reconnection). Gaps were treated as missing data and characters treated as multistate and unordered. Distance analyses were performed by neighbour joining (NJ) using the general time reversible (GTR) model. The optimal model for the maximum likelihood analyses (ML) was found with 99% level of significance in Modeltest version 3.7 [32]. ML analyses were done by heuristic searches with 10 random addition replicates and the TBR branch-swapping algorithm. One thousand bootstrap replicates were performed in MP and NJ and 100 in ML. Bayesian analyses were done using MrBayes 3.1.2 [33]. The analyses using four chains were run for 1,200,000 generations, the temperature set to 0.2. Sample frequency was set to 100 and the number of burn-in generations was 3,000.

## Results

The results of the molecular analyses showed clustering of the strains into five groups, which corresponded to the different morphotypes observed among the strains. Two of the morphotypes corresponded to *C*. *decipiens* and *C*. *mitra*, which are emended below. The other three are described as new species, i.e. *C*. *elegans* sp. nov., *C*. *laevisporus* sp. nov. and *C*. *mannaii* sp. nov.

### Morphological studies

**Fig 1:**
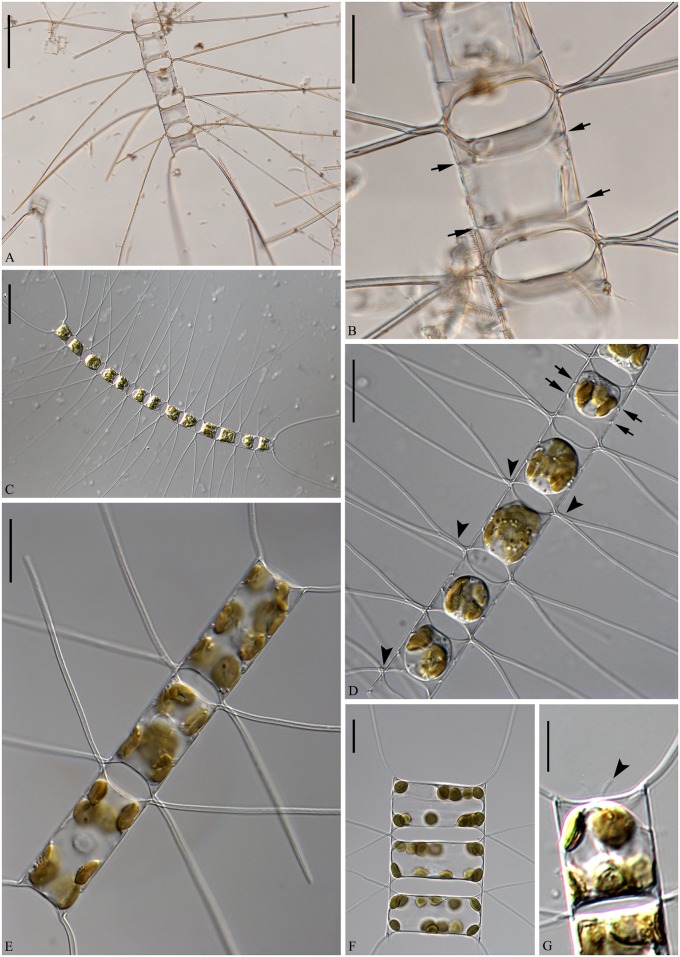
LM of *Chaetoceros decipiens*. A, C, E, F: Chain views of lectotype material MIC5366 (A) and strains P10E5 (C), P14B3B (E), D12 (F). B and D: Detail of chains showing constrictions (arrows) between the mantle and the girdle bands, and the fused extensions of sibling setae, which are short, longer or even absent (arrowheads); lectotype material MIC5366 (B) and strain P10E5 (D). G: Chain view of strain D10, note the V-shaped protrusion located centrally on the terminal valve (arrowhead). A and C: scale bars, 50 μm. B, D, E, F: scale bars, 20 μm. G: scale bar, 10 μm.

**Fig 2:**
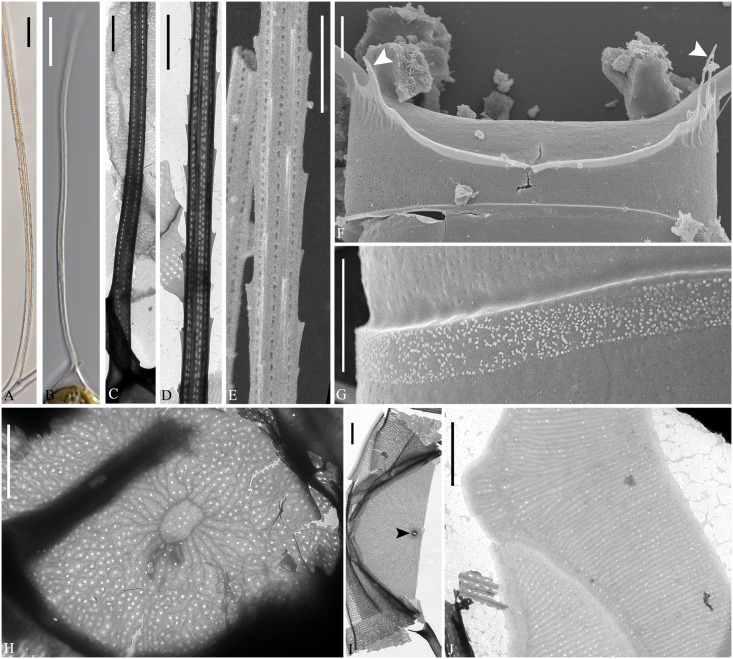
*Chaetoceros decipiens*. LM (A and B), TEM (C, D, H-J) and SEM (E-G). A-E: Seta structure of lectotype MIC5366 (A), strains P14B3B (B) and D10 (C–E), showing the 4–6 sided seta with poroids and small spines. F: Terminal valve with fringes (arrowheads); strain D10. G: Silica warts on the basal ring of the mantle; strain D10. H: Annulus, costae and poroid pattern on intercalary valve; strain P10E5. I: Terminal valve showing rimoportula without external process (arrowhead); strain D10. J: Girdle bands; strain D10. A and B scale bars, 10 μm. C–J scale bars, 2 μm.

**Fig 3:**
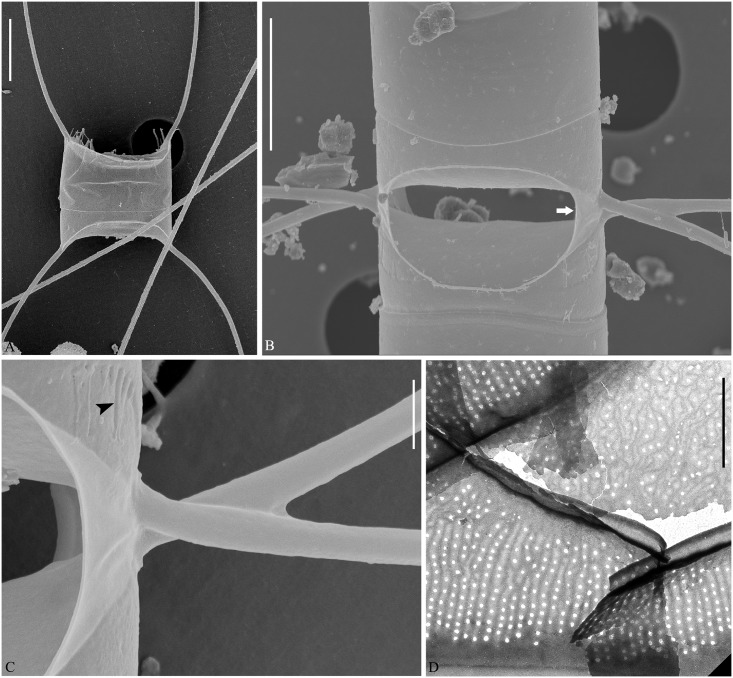
*Chaetoceros decipiens*. Strain D10, SEM (A–C) and TEM (D). A: Solitary cell with silica fringes. B: Intercalary cells with overlapping silica membrane (arrow). C: Detail of fused seta bases, silica membrane and fringes on the mantle (arrowhead). D: Rows of poroids on the mantle. A and B scale bars, 10 μm. C and D scale bars, 2 μm.

***Chaetoceros decipiens*** Cleve 1873, p. 11, Pl. I, fig 5a & 5b, emend. Li, Boonprakob, Moestrup & Lundholm Figs 1–3, 20E and 20F and S1 and S2 Figs.

Synonym: *Chaetoceros grunowii* Schütt 1895

Lectotype designated here. A holotype was not chosen by Cleve. We have chosen slide MIC5366 in P.T. Cleve’s collection, Stockholm, Sweden as the lectotype. The slide is labelled “Atlanten ytan 27/5-71 Lat 60°25 Long 19°50 ThM Fries”. Figs 1A, 1B and 2A illustrate the lectotype.

Examination of the type material revealed the morphology to be in agreement with Cleve’s description and with our strains identified as *C*. *decipiens*. The Cleve material from the North Atlantic and the Davis Strait looked similar. Most prominently some sibling setae were found to fuse for a longer distance; however, in others no fusion was seen. A very delicate striation of the setae was observed, in agreement with Cleve [7]. We did not at first observe the striation in our cultured material, but a closer examination showed a similar and extremely delicate striation.

Type locality: North Atlantic 60°25’N 19°50’W

Epitype designated here: Glutaraldehyde-fixed material of strain P10E5 isolated from the Norwegian Sea (67,9050N, 4.3238W). The material has been deposited at the Natural History Museum of Denmark, Copenhagen (C-A-92068). Fig 1C and 1D illustrate the epitype. A sequence of D1-D3 LSU rDNA represents the epitype (Genbank accession number KX065223).

Emended description: Chains are usually straight, sometimes slightly arc-shaped curved (Fig 1A, 1C, 1E and 1F). In broad girdle view, the cells are quadrangular or rectangular (Fig 1A, 1B and 1D–1G) sometimes with the apical axis longer than the pervalvar axis (Fig 1F), sometimes the reverse (Fig 1B, 1D, 1E and 1G). Solitary cells also occur (Fig 3A). Several (3–12) chloroplasts are present in each cell (Fig 1E and 1F). In valve view, valves are broadly elliptical to round-oval; the valve face is saddle-shaped (Figs 2F and 3B), the central region slightly raised and higher than the valve face margin. A silica rib along the valve face margin is broadly arcshaped (Figs 2F and 3B). On the valve face, the costae diverge while anastomosing from a central annulus, with solitary poroids scattered between the costae (Fig 2H). The intercalary valve corners touch those of the adjacent cells (Fig 1B and 1D–1F). The apertures are variable, narrow slit-like oval to hexagonal (Fig 1B and 1D–1G).

Setae are stiff and extend from the corners of the cell (Fig 1A–1F). All setae of a chain are located more or less in the apical plane (Brunel group I) [34] (Fig 1A–1D), sometimes diverging very slightly from the apical plane (Fig 1E). Sibling setae cross over just outside the chain border and may fuse for a shorter or longer distance (Figs 1A–1D, 3B and 3C). The setae lack a basal part (Figs 1B, 1D and 3C). The extent of fusion varies even within a single chain and between the two sides of adjacent cells (Fig 1D, arrowheads, Fig 3B). The terminal setae diverge, forming an open U (Figs 1A, 1C, 1F and 3A). On the intercalary valve, a silica membrane of variable size is present at the margin of the apertures, forming a continuation of the marginal silica rib. The membranes of sibling cells may overlap to form a junction between the cells (Fig 3B, arrow, Fig 3C). Silica fringes are present on the mantle below the membranes (Fig 3C, arrowhead) and are more distinct on the terminal valves, sometimes with long protuberances (Fig 2F, arrowheads, Fig 3A). Four to six rows of poroids and spines are arranged in longitudinal rows along the setae (Fig 2C–2E). The poroids are round-oval (Fig 2C–2E), 0.3±0.1 μm long in size and with a density of 19.0±6.7 poroids in 10 μm (n>50). Sometimes a striation is visible under LM (Fig 2A), sometimes not (Fig 2B), not reflecting poroid density, but more or less similar to the density of the spines (Table 1). All setae have the same structure.

**Table 1:** Morphological characters for differentiating *C*. *decipiens*, *C*. *elegans*, *C*. *laevisporus*, *C*. *mannaii*, *C*. *mitra* and *C*. *lorenzianus*.

Character	*C*. *decipiens*	*C*. *elegans*	*C*. *laevisporus*	*C*. *mannaii*	*C*. *mitra*	*C*. *lorenzianus* type material
Seta poroid shape	round-oval	tear-shaped	round-oval	oval	round-oval	round
Seta poroid size (μm)	0.1–0.6	0.2–1.3	0.3–0.9	0.4–1.2	0.1–0.3	n.d.
	(0.3±0.1)	(0.5±0.2)	0.6±0.1	0.7±0.2	0.2±0.1	
Seta poroid number in 10 μm	14–38	7–29	11–17	11–15	30–56	5–9
	(19.0±6.7)	17.8±5.4	13.8±1.9	12.3±1.6	39.8±7.4	7.2±1.7
Brunel group	I	I	I	I	II	I
Fusion of seta bases	present/ absent	absent	absent	absent	absent	present
Resting spore	unknown	two branching processes	smooth	unknown	two branching processes	two branching processes?
Aperture shape	oval-hexagonal	rounded quadrangular	oval peanut-shaped	hexagonal	oval-hexagonal, peanut shaped	oval-hexagonal
Aperture/pervalvar axis index	0.5	0.5	0.4	0.3	0.2	0.5–1.0
Basal part of setae	lacking	present	lacking	present	lacking	present
Poroids on valve face	yes	yes	no	no	no	n.d.
External tube of rimoportula	no	short	no	distinct	no	n.d.
Apical axis (μm)	7.8–64.3	11.7–39.7	22.4–46.3	5.7–12.9	16.5–23.8	20–43
	(29.5±14.7)	(26.4±10.6)	(32.7±7.9)	(10.3±2.1)	(20.6±2.0)	
Pervalvar axis (μm)	7.8–78.9	8.9–42.2	13.2–42.5	8.4–29.6	28.1–48.2	n.d.
	(18.5±10.9)	(18.6±8.1)	(21.6±6.4)	(17.0±5.2)	(35.6±5.2)	
Aperture in pervalvar axis (μm)	3.3–15.1	4.4–14.5	6.3–12.0	4.6–5.6	2.9–10.0	n.d.
	(9.0±2.8)	(10.1±2.2)	(9.6±1.5)	(5.1±0.4)	(6.7±1.3)	

n.d. indicates no data available

A single rimoportula without an external tube is situated centrally on the terminal valve (Fig 2I, arrowhead). No processes were observed on the intercalary valves (Fig 2H). Sometimes, V-shaped non-silicified protrusions can be seen in LM centrally on the terminal valves (Fig 1G, arrowhead). In LM, a constriction is visible at the border between the mantle and the girdle bands (Fig 1B and 1D, arrows), and the mantle occupies approximately one third of the pervalvar axis. The mantle has narrow parallel rows of costae separated by single rows of poroids (Fig 3D). Silica warts are present on the basal ring of the mantle (Fig 2G).

The girdle bands have parallel costae separated by single rows of scattered poroids (Fig 2J). The apical axis of the valve is 7.8–64.3 μm long, the pervalvar axis 7.8–78.9 μm long, the length of the aperture in the pervalvar axis 3.3–15.1 μm (n>100). No resting spores were found.

Sexual reproduction was observed, and was thus homothallic (S1 and S2 Figs). Auxospores adhered to the girdle of the mother cell (S1 and S2 Figs, arrows). New daughter cells achieved a larger apical axis of the valves (S2 Fig).

Geographical distribution: Davis Strait, North Atlantic [7]; Disko Bay, Greenland (April, present study); Denmark Strait; Beaufort Sea; Norwegian Sea; Denmark (April, present study; most of the year with a maximum in spring [9]); Narragansett Bay of Rhode Island [10]; Gulf of Naples, Italy ([35], present study); Peter the Great Bay, Sea of Japan [13]; Japanese coast [36]; Pacific coast of Mexico [12]; southern Gulf of Mexico [15]; Daya Bay, south China (December, present study).

**Fig 4:**
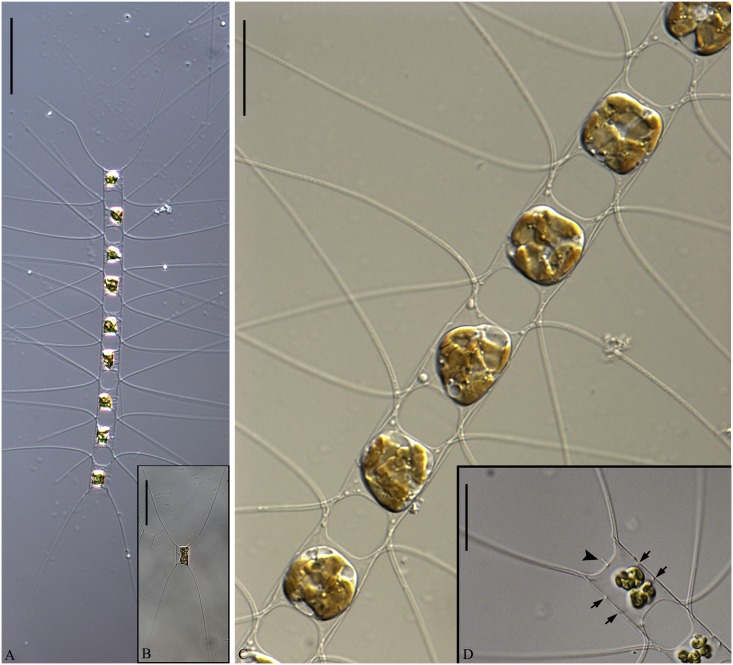
LM of *Chaetoceros elegans* sp. nov. Strain YL7. A: Chain view displaying seta divergence in the apical plane. B: Solitary cell. C: Chain view demonstrating large apertures and chloroplasts. D: End of chain showing constrictions (arrows) between the mantle and the girdle, and V-shaped protrusion (arrowhead) located centrally on terminal valve. A and B scale bars, 50 μm. C and D scale bars, 20 μm.

**Fig 5:**
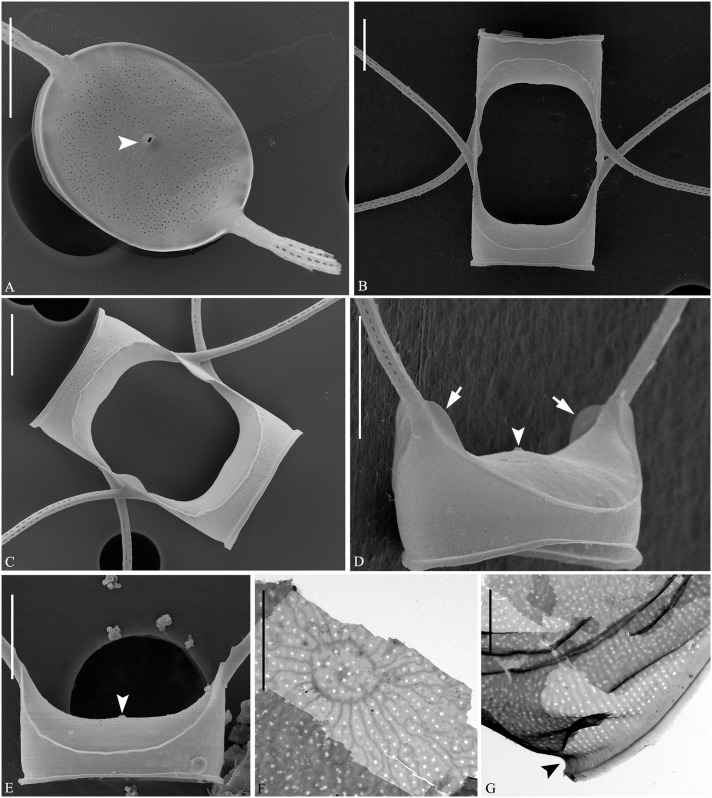
*Chaetoceros elegans* sp. nov. SEM (A–E) and TEM (F and G). A: Terminal valve view with external process of rimoportula and poroids on valve face; strain MC1048. B and C: Intercalary cells demonstrating large apertures and valve faces; strain M1 (B), strain MC1048 (C). D and E: Terminal valves with central processes (arrowheads) and silica ear-like structures (arrows in D) in strain YL7. F: Annulus, costae and poroid pattern near intercalary valve centre; strain YL7. G: Parallel rows of poroids on the mantle; arrowhead indicates ring-shaped constriction; strain YL7. A–E scale bars, 5 μm. F and G scale bars, 2 μm.

**Fig 6:**
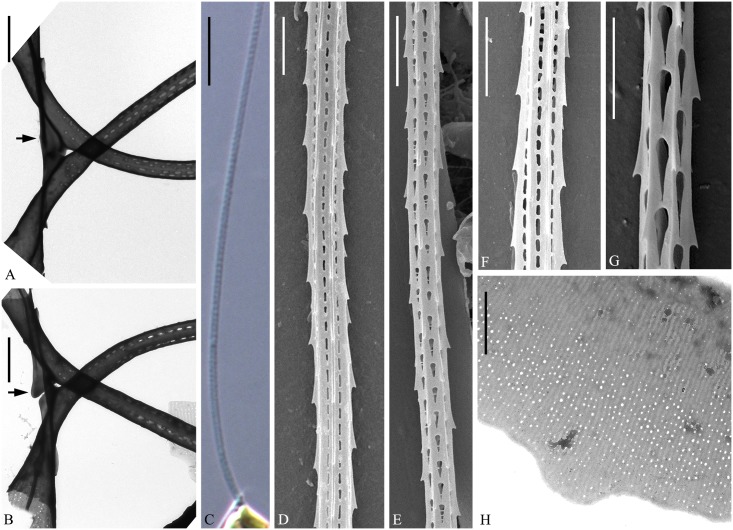
*Chaetoceros elegans* sp. nov. LM (C), TEM (A, B, H) and SEM (D–G). A and B: Overlapping ear-like structures (arrows) and small gap between the crossing bases of sibling setae in strain YL7. C: Terminal seta with partly visible poroids in LM; strain YL7. D and E: Seta structure showing elongated poroids (D, strain Ch12A1) and tear-shaped poroids (E, strain M1) and F and G: Detail of setae poroids; strain Ch12A1 (F) and strains MC785 (G). H: Girdle band; strain YL7. All scale bars are 2μm, except 10 μm in C.

**Fig 7:**
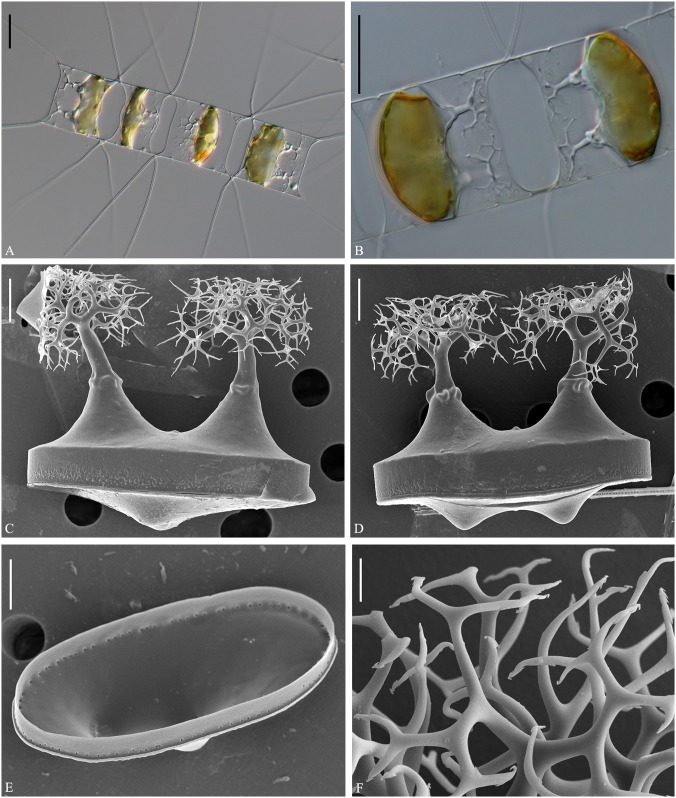
Resting spores of *Chaetoceros elegans* sp. nov. LM (A and B) and SEM (C–F). A and B: Resting spores within mother cells in a chain; strain MC785. C and D: Released resting spores, showing two elongated elevations with dichotomous branching processes distally on the primary valve face and one (C) or two bulges (Fg D) on the secondary valve face; strain Ch12A1. E: Internal view of secondary valve with a ring of marginal punctae; strain Ch12A1. F: Hooks on the distal tips; strain MC785. A and B scale bars, 20 μm. C–E scale bars, 5 μm. F scale bar, 1 μm.

***Chaetoceros elegans*** Li, Boonprakob, Moestrup & Lundholm **sp. nov**. Figs 4–7

Formal diagnosis: Straight chains or solitary cells. Four to ten chloroplasts typically present in each cell. Apical axis 11.7–39.7 μm. Pervalvar axis 8.9–42.2 μm. Aperture in pervalvar axis 4.4–14.5 μm. Cells quadrangular in girdle view. Saddle-shaped valve face. Central annulus, diverging costae and scattered poroids on valve face, continuing onto mantle. Silica rib on the valve face edge. A rimoportula present on the terminal valve. Furrow above the basal ring of mantle. Large and rounded, quadrangular-rectangular apertures. Setae in the apical plane. Basal part of setae extend in the pervalvar direction. Sibling setae cross over outside chain border without fusing. Terminal setae diverge in direction of chain. Silica ear-like structures present on base of setae. Four to six rows of poroids and spines on the four-six sided setae. Tear-shaped to elongate poroids on setae, ca. 0.5±0.2 μm in size and 17.8±5.4 poroids in 10 μm. Several bands, each band with parallel costae and scattered poroids. Resting spore with smooth surface. The primary valve extends into two elongated elevations with dichotomous branching processes. The secondary valve with one or two bulges. The angle of the outer slope of the elevation is acute. Length of elevation is 1–2 times longer than the branching processes.

Holotype: Glutaraldehyde-fixed material of strain YL7 deposited at the Natural History Museum of Denmark, Copenhagen (C-A-92069). Figs 4A–4D, 5D–5G and 6A–6C, H illustrate the holotype. A sequence of D1-D3 LSU rDNA represents the holotype (Genbank accession number KX065232).

Type locality: Dapeng Bay, Guangdong Province, P. R. China.

Etymology: referring to the characteristic very elegant overall look of the chains and the resting spores.

The chains are straight and stiff (Fig 4A). Cells are quadrangular in broad girdle view (Fig 4A–4D). Solitary cells also occur (Fig 4B). Typically four to ten chloroplasts are present within each cell (Fig 4C). The valves are broadly elliptical to round-oval (Fig 5A) with a saddle-shaped valve face, as the central region is slightly raised (Fig 5B–5E). The valve face edge is broadly arc shaped and marked by an elevated silica rib (Fig 5B–5E). On the valve face, costae diverge from a central annulus, with poroids scattered in between (Fig 5F). A constriction is visible at the border between the mantle and the girdle bands (Fig 4D, arrows). The mantle occupies nearly one third of the pervalvar axis, and is ornamented with narrow parallel rows of costae interspersed by single rows of poroids (Fig 5G). A ring-shaped furrow is present above the basal ring of the mantle (Fig 5B–5E and 5G arrowhead). Apertures are large and rounded quadrangular-rectangular (Figs 4C, 5B and 5C).

All setae of a chain are located in the apical plane (Brunel group I) (Fig 4A) [34]. The setae protrude from the elongated corners of the cell (Figs 4C, 5B and 5C). The basal parts of the setae extend initially in approximately the direction of the pervalvar axis, before curving and crossing over (Fig 5B and 5C). Sibling setae diverge at an acute angle to each other, and cross over just outside the chain border without fusing (Figs 4C, 5B and 5C). Intercalary setae near the ends of the chain are directed slightly more in the direction of the chain ends (Fig 4A). The two terminal setae diverge slightly, continuing more or less in the direction of the chain (Fig 4A and 4B). On the terminal valve, two silicified, ear-like structures project from the base of the setae, one on each side (Fig 5D, arrows), forming a continuation of the narrow silica rib on the valve edge. On the intercalary valves, these ‘ears’ of sibling cells overlap and form a junction between the cells, in some cases fusing (Figs 5B, 5C, 6A and 6B, arrows). A small gap is sometimes seen between the crossing bases of sibling setae and the overlapping, ear-like structures (Fig 6A and 6B). The setae are four to six-sided, with four to six longitudinal rows of poroids and spines arranged alternatingly on the setae (Fig 6D–6G). The seta poroids are tear shaped (Fig 6D–6G), 0.5±0.2 μm long with a density of 17.8±5.4 poroids in 10 μm (n>70) (Table 1). The poroids are visible in LM (Fig 6C). Poroids are smaller, oval and less numerous near the base of the setae (Fig 6A and 6B). All setae have the same structure.

A single rimoportula with a short external tube is situated centrally on the terminal valve (Fig 5A, 5D and 5E), while processes are absent on the intercalary valves (Fig 5B, 5C and 5F). In LM, a V-shaped non-silicified protrusion is visible centrally on the terminal valve (Fig 4D, arrowhead). Several open girdle bands are present, each band ornamented with parallel costae, which are separated by single rows of scattered poroids (Fig 6H). The apical axis is 11.7–39.7 μm long, the pervalvar axis 8.9–42.2 μm long, the pervalvar axis including basal parts 17.1–31.9 μm long, the length of the aperture in the pervalvar axis 4.4–14.5 μm (n>80).

The resting spores are located centrally in the mother cells, touching the bands and sometimes the valves of the mother cell (Fig 7A and 7B). The surface of the resting spore is mainly smooth (Fig 7C and 7D). The primary valve extends into two elongated elevations with dichotomous branching processes, and one or two bulges are present on the secondary valve face (Fig 7A–7D). The elevations are 32.5–48.0 μm long, the branching processes 5.5–14.2 μm long, and the apical axis 32.5–48.0 μm. The angle of the outer slope of the elevation is acute (Fig 7C and 7D). Length of elevation is 1–2 times longer than the branching processes (Table 2). Sometimes, several silica bulges are located at the bases of the two processes (Fig 7C and 7D). Each process branches into a tree-like structure with the distal tips pointed and possessing one or several hooks (Fig 7F). A single ring of puncta is located near the margin of the secondary valve mantle (Fig 7E).

**Table 2:** Morphological characters of resting spores of *C*. *elegans* and C. *mitra*.

Characters	*C*. *elegans*	*C*. *mitra*
Apical axis (μm)	36.5±3.4	18.1±2.3***
	(32.5–48)	(14.6–21.4)
Pervalvar axis of primary valve (μm)	13.2±2.3	25.4±3.9***
	(9.0–18.5)	(21.8–36.2)
Length of branching processes (μm)	9.1±2.2	8.3±1.5
	(5.5–14.2)	(5.7–11.4)
Outer slope of elevation	Acute angle	Almost straight
Joining of two elevations	Near mantle	Halfway
Secondary valve bulges	1–2	1–2
Elevations/branching processes	1.6±0.3	3.2±0.8***
	(1.1–2.1)	(2.2–4.5)

*** indicates statistical difference (p<0.0001)

Geographical distribution: Japanese coast (as *C*. *decipiens* in [36]); Gulf of California (as *C*. *lorenzianus* in [12]); New Brunswick Canada (present study); Concepción, Chile (October, present study); Dapeng Bay, south China (August, present study); Mannai Island, Thailand (June, present study).

**Fig 8:**
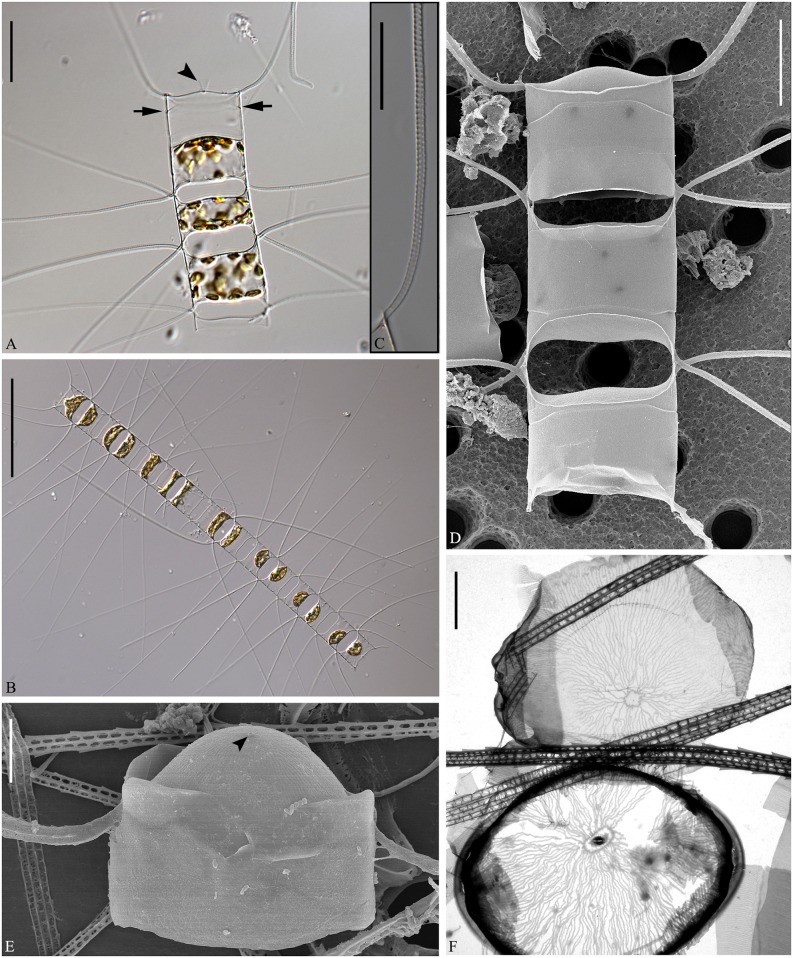
*Chaetoceros laevisporus* sp. nov. LM (A–C), SEM (D and E) and TEM (F). Fig A: Part of a chain showing constrictions (arrows) between mantle and girdle, and V- shaped protrusion (arrowhead) located centrally on the terminal valve; strain N7. B: Dividing chain showing seta divergence; strain DY1. C: Structure of terminal seta; strain DY1. D: Chain in girdle view showing cells and apertures; strain N7. E: Terminal valve with rimoportula without external process (arrowhead); strain N7. F: Valve views of intercalary (upper) and terminal valves (below); strain N7. A, C, D scale bars, 20 μm. B scale bar, 100 μm. E and F scale bars, 5 μm.

**Fig 9:**
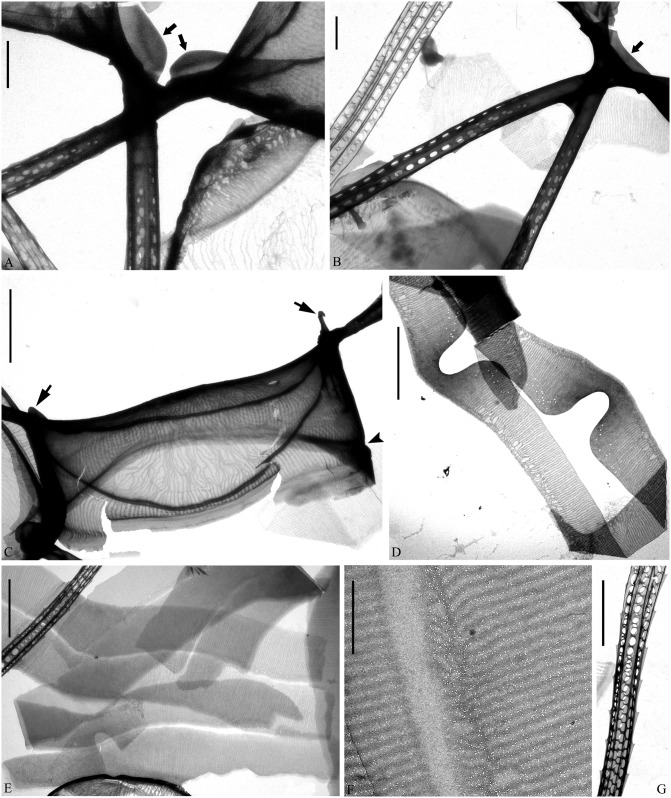
TEM of *Chaetoceros laevisporus* sp. nov. Strain N7. A, B: Non-overlapping (arrows in A) and overlapping ears (arrow in B) between sibling cells. C: Terminal valve with silica fringe (arrows) and constriction (arrowhead). D and E: Open girdle bands. F: Detail of girdle band with pores. G: Seta with round-oval poroids. A, B scale bars, 2 μm. C–E, G scale bars, 5 μm. F scale bar, 1 μm.

**Fig 10:**
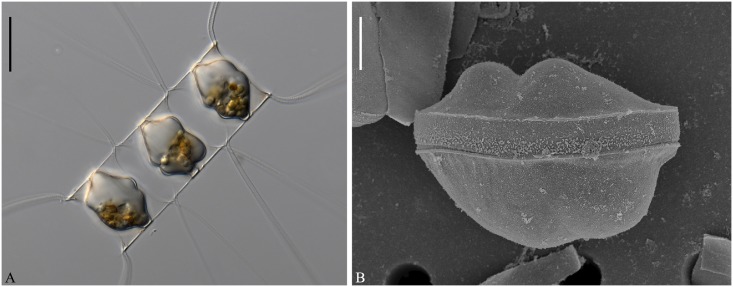
Resting spores of *Chaetoceros laevisporus* sp. nov. LM (A) and SEM (B). Resting spores within mother cells of a chain (A) and single, released resting spore (B); strain DY1. A scale bar, 20 μm. B scale bar, 5 μm.

***Chaetoceros laevisporus*** Li, Boonprakob, Moestrup & Lundholm **sp. nov**. Figs 8–10

Formal diagnosis: Straight chains. Several chloroplasts in each cell. Apical axis 22.4–46.3 μm. Pervalvar axis 13.2–42.5 μm. Aperture in pervalvar axis 6.3–12.0 μm. Cells rectangular in girdle view. Saddle-shaped valve face. Costae diverge from a central annulus on the valve face, continuing on to the mantle. Silica rib on the valve edge. A rimoportula present on the terminal valve. A furrow located above the basal ring of the mantle. Oval-peanut shaped apertures. Setae in the apical plane. Valve corners on sibling valves touch each other. Sibling setae cross over just outside the chain border. Terminal setae diverge in the direction of the chain. Silica ear-like structures and fringes present on the base of setae. Four to six rows of poroids and spines on the setae. Round-oval setae poroids, 0.6±0.1 μm in size, with 13.8±1.9 poroids in 10 μm. Several bands with parallel costae and separated poroids. Smooth resting spores with two conical elevations on the primary valve and one or two on the secondary valve.

Holotype: Glutaraldehyde-fixed material of strain N7 deposited at the Natural History Museum of Denmark, Copenhagen (C-A-92070). Figs 8A, 8D–8F and 9A–9G illustrate the holotype. A sequence of D1-D3 LSU rDNA represents the holotype (Genbank accession number KX065240).

Type locality: Mannai Island, Rayong Province, Thailand.

Etymology: laevis (Lat.): smooth, the resting spores are smooth without extensions.

The chains are straight and stiff (Fig 8A, 8B and 8D). In broad girdle view, cells are rectangular, the apical axis usually longer than the pervalvar axis (Fig 8A and 8B). Several chloroplasts (more than ten) are present within each cell (Fig 8A). The valves are broadly elliptical to round-oval (Fig 8F). The valve face is saddle shaped, as the central region is slightly raised (Fig 8D). The valve face edge is broadly arc shaped and marked by an elevated silica rib (Fig 8D). On the valve face, costae diverge from a central annulus, without poroids between the costae (Figs 8F and 9C). In LM, a constriction is visible at the border between the mantle and the girdle bands, and the mantle occupies approximately one third of the pervalvar axis (Fig 8A, arrows). The mantle is ornamented with narrow, parallel rows of costae (Fig 9C). A circular furrow is present above a basal ring of the mantle (Fig 9C, arrowhead). Valve corners on intercalary valves are elevated and almost touch those of adjacent cells (Fig 8A and 8D). Apertures are oval-peanut shaped (Fig 8A, 8B and 8D).

Setae of a chain are situated more or less in the apical plane (Brunel group I) (Fig 8B), sometimes diverging very slightly from the apical plane (Fig 8A). The intercalary setae are straight or slightly curved, those near the ends curving more towards the ends (Fig 8B). The setae vary greatly in length (Fig 8B). They protrude from the corners of the cell, and sibling setae cross over just outside the chain border, without any fusion (Fig 8A and 8D). An ear-like structure is present at the seta base of intercalary valves near the marginal border of the aperture (Fig 9A, arrows). The ‘ears’ sometimes form a junction between sibling cells (Fig 9B, arrow). The two terminal setae diverge slightly, continuing more or less in the direction of the chain (Fig 8A and 8B). The setae are four to six-sided, and four to six longitudinal rows of poroids and spines are arranged alternatingly on the setae (Fig 9G). The seta poroids are round-oval (Fig 9G), 0.6±0.1 μm μm long, with 13.8±1.9 poroids in 10 μm (n>25), visible in LM (Fig 8C)(Table 1). Poroids near the seta bases are smaller and more scattered (Fig 9B). All setae have the same structure.

A single rimoportula, which lacks an external tube, is situated centrally on the terminal valve (Fig 8E, arrowhead, Fig 8F, lower valve). In LM, a V-shaped non-silicified protrusion is visible centrally on the terminal valves (Fig 8A, arrowhead). Processes are absent on the intercalary valves (Fig 8F, upper valve). Siliceous fringes are present near the terminal seta base (Fig 9C, arrows). Several open girdle bands are present (Fig 9D), each with parallel costae separated by two, occasionally three, rows of scattered pores, in addition to larger poroids (Fig 9E and 9F). The apical axis is 27.7–34.2 μm long, the pervalvar axis 13.2–42.5 μm long, and the length of the aperture in the pervalvar axis measures 6.3–12.0 μm (n>80).

The resting spores are located centrally in the mother cells, touching both valves and bands of the mother cell (Fig 10A). The spore surface is smooth with two conical elevations on the primary valve and one or two on the secondary valve (Fig 10A and 10B).

Geographical distribution: Daya Bay, south China (December, present study); Mannai Island, Thailand (December, present study), Gulf of Panama (as *C*. cf. *lorenzianus* in [35]).

**Fig 11:**
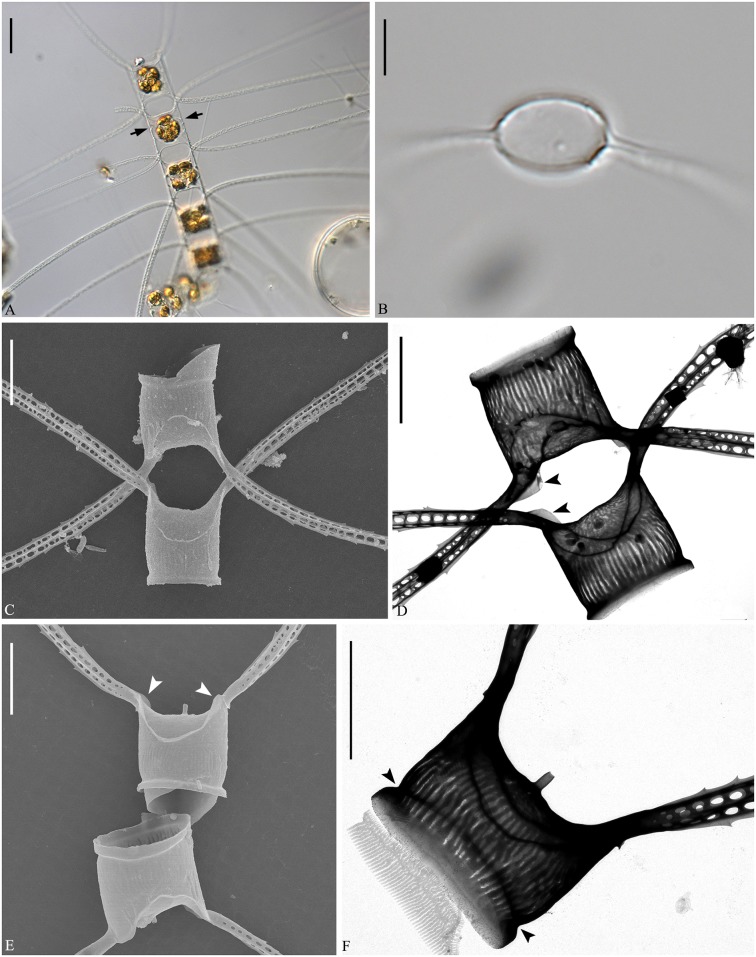
*Chaetoceros mannaii* sp. nov. LM (A and B), SEM (C and E) and TEM (D and F); strain N1. A: Straight chain showing seta divergence and constrictions (arrows) between the mantle and the girdle. B: Oval valve face. C and D: Intercalary cells, with ear-like structures (arrowheads in D) at the bases of setae in heavily silicified frustule. E and F: Terminal valves, with ear-like structures at the seta bases (arrowheads in E) and distinct constriction above the ring (arrowheads in F). A scale bar, 20 μm. B–F scale bars, 5 μm.

**Fig 12:**
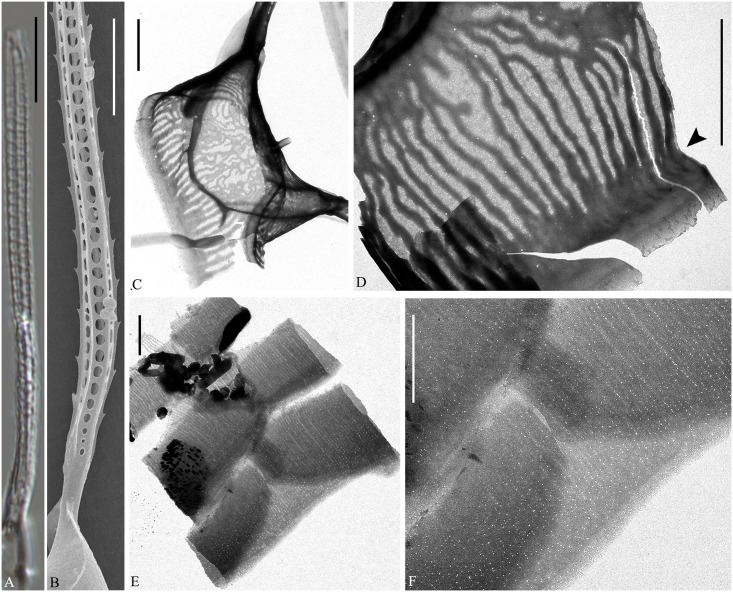
*Chaetoceros mannaii* sp. nov. LM (A), SEM (B) and TEM (C–F); strain N1. A and B: Setae with oval poroids. C: Terminal valve showing valve structure and rimoportula with external projection. D: Poroids and costae on the mantle. E and F: Girdle bands with poroids. A scale bar, 10 μm. B scale bar, 5 μm. C–F scale bars, 2 μm.

***Chaetoceros mannaii*** Boonprakob, Li, Moestrup & Lundholm **sp. nov**. Figs 11 and 12

Formal diagnosis: Short straight chains or solitary cells. Several chloroplasts in each cell. Apical axis 5.7–12.9 μm. Pervalvar axis 8.4–29.6 μm. Aperture in pervalvar axis 4.6–5.6 μm. Cells rectangular in girdle view. Saddle-shaped valve face. Heavily silicified frustule. Robust diverging costae on the valve face, continuing onto the mantle. Silica rib on the valve face edge. A rimoportula, with long external process on the terminal valve. The mantle occupies one fourth of the pervalvar axis. A furrow is present above the basal ring of the mantle. Hexagonal apertures. Setae in or slightly diverging from the apical plane. Short basal part present, sibling setae cross over outside chain border. Terminal setae diverge in the direction of the chain. Silica ear-like structures present on the base of setae. Four to six rows of poroids and spines on the setae. Large oval setae poroids, 0.7±0.2 μm in size, 12.3±1.6 poroids in 10 μm. Several bands with parallel costae and scattered pores.

Holotype: Glutaraldehyde-fixed material of strain N1 deposited at Natural History Museum of Denmark, Copenhagen (C-A-92071). Figs 11 and 12 illustrate the holotype. A sequence of D1-D3 LSU rDNA represents the holotype (Genbank accession number KX065246).

Type locality: Mannai Island, Rayong Province, Thailand.

Etymology: from Mannai Island, Thailand.

Short straight chains are typical (Fig 11A), but solitary cells also occur. Several chloroplasts (4–10) are present within each cell (Fig 11A). In broad girdle view, cells are rectangular (Fig 11A). In valve view, valves are broadly elliptical to round-oval (Fig 11B). The valve face is saddle shaped, as the central region of the valve face is slightly raised (Fig 11C and 11D). The valve face edge is broadly arc shaped and marked by an elevated silica rib (Fig 11C–11E). Robust costae diverge from the centre of the valve face, without poroids between the costae (Figs 11F and 12C), and continue onto the mantle as robust parallel longitudinal ribs (Fig 12D). In LM, a constriction is visible at the border between the mantle and the girdle bands (Fig 11A, arrows), and the mantle occupies ca. one fourth of the pervalvar axis. The basal ring of the mantle is heavily silicified, with a distinct furrow above the ring (Figs 11F and 12D, arrowheads). The apertures are hexagonal (Fig 11A, 11C and 11D).

Setae of a chain seem to be located more or less in the apical plane (Brunel group I), or sometimes slightly diverging from the apical plane (Fig 11B). The intercalary setae are straight or slightly curved (Fig 11B). The setae protrude from the elevated corners of the cell (Fig 11C). Sibling setae cross over just outside the chain border, with short basal parts present (Fig 11C and 11D). Terminal setae diverge in an acute to 90 degrees angle (Fig 11A, 11E and 11F). Silicified ear-shaped structures are located at the base of the setae on both the intercalary and terminal valves (Fig 11D and 11E, arrowheads). The ‘ears’ form a continuation of the narrow silica rib on the valve face edge (Fig 11E). In the intercalary valves, the ears of sibling setae do not appear to overlap (Fig 11C and 11D). Setae are four-six sided with four to six longitudinal rows of poroids and spines arranged alternatingly on the setae (Fig 12B). The seta poroids are oval (Fig 12B), 0.7±0.2 μm long and with 12.3±1.6 poroids in 10 μm (n = 20), readily visible in LM (Figs 11A and 12A)(Table 1). Those near the seta base are slightly smaller and more scattered (Fig 12B). All setae have the same structure.

A single rimoportula with a long external tube is situated centrally on the terminal valve (Figs 11E, 11F and 12C). Processes are absent on the intercalary valves (Fig 11C and 11D). Several open girdle bands are present (Fig 12E), each with parallel costae separated by one row of scattered pores (Fig 12F). The apical axis is 6.7–12.9 μm long, the pervalvar axis 8.4–29.6 μm long, the length of the aperture in the pervalvar axis 4.6–5.6 μm (n = 20). No resting spores were found.

Geographical distribution: Peter the Great Bay, Sea of Japan (as *C*. *lorenzianus* in [13]; Gulf of California (as *C*. *lorenzianus* in [12]; Sinaloa, Mexico (present study); near Mannai Island, Thailand (present study).

**Fig 13:**
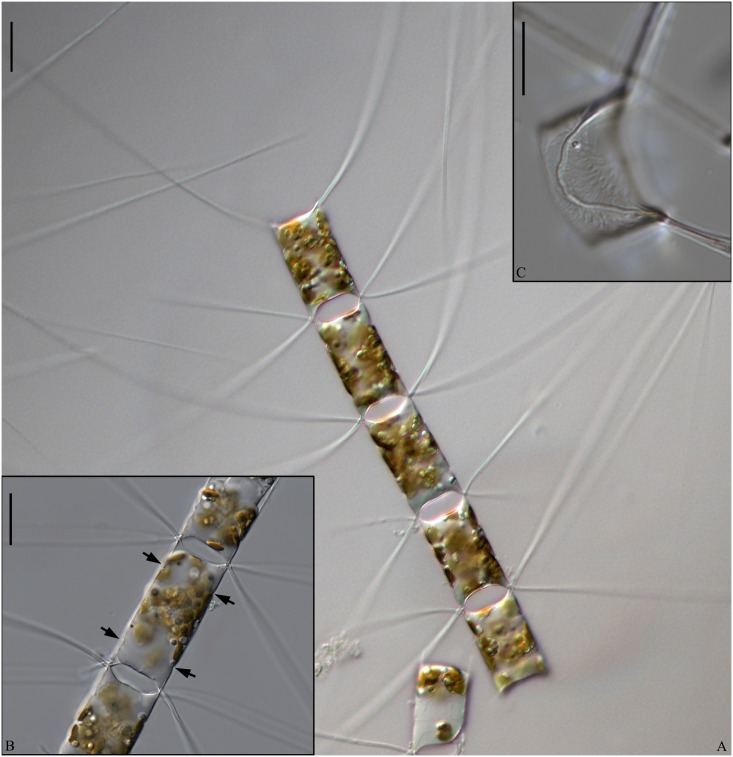
LM of *Chaetoceros mitra*. Strain P10A1. A: Straight chain showing seta divergence and apertures. B: Detail of chain showing constrictions (arrows) between mantle and girdle. C: Valve structure of terminal valve. A and B scale bars, 20 μm. C scale bar, 10 μm.

**Fig 14:**
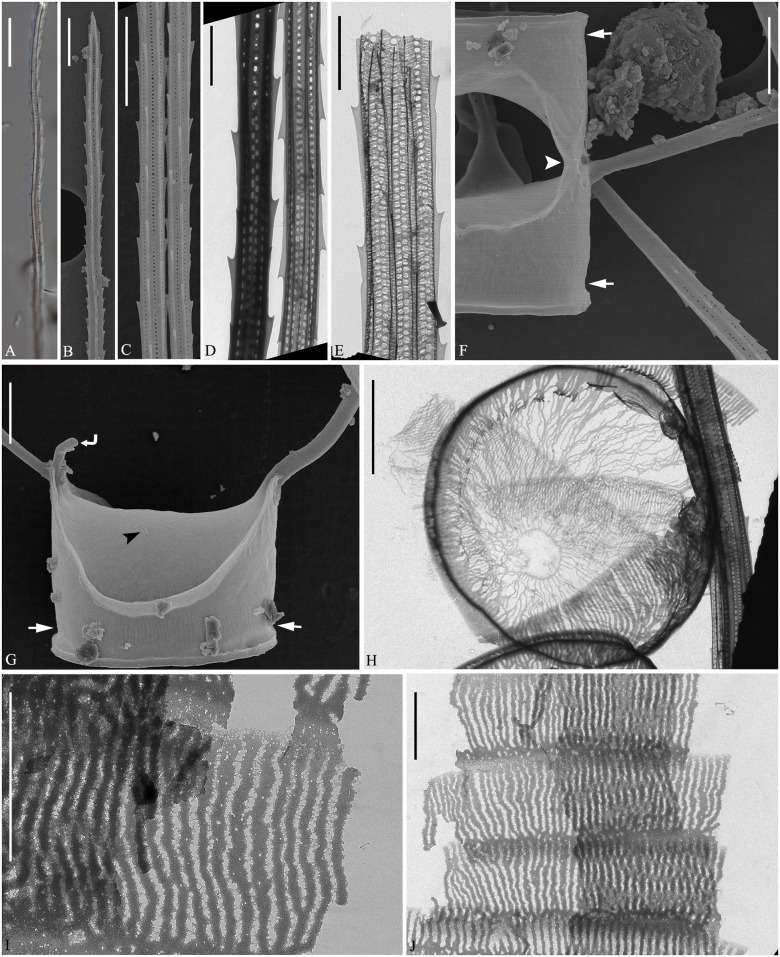
*Chaetoceros mitra*. LM (A), SEM (B, C, F, G) and TEM (D, E, H–J); strain P10A1. A–E: Setae showing round-oval poroids and spines, using different microscopy techniques. F: Intercalary valves showing wing-like structures (arrowhead), and furrows above the basal ring of mantle (arrows). G: Terminal valve showing rimoportula without external tube (arrowhead), furrow above the basal ring of mantle (arrows) and fringe (curved arrow). H: Intercalary valve face. I and J: Girdle bands. A scale bar, 10 μm. B, C, F–H scale bars, 5 μm. D, E, I, J scale bars, 2 μm.

**Fig 15:**
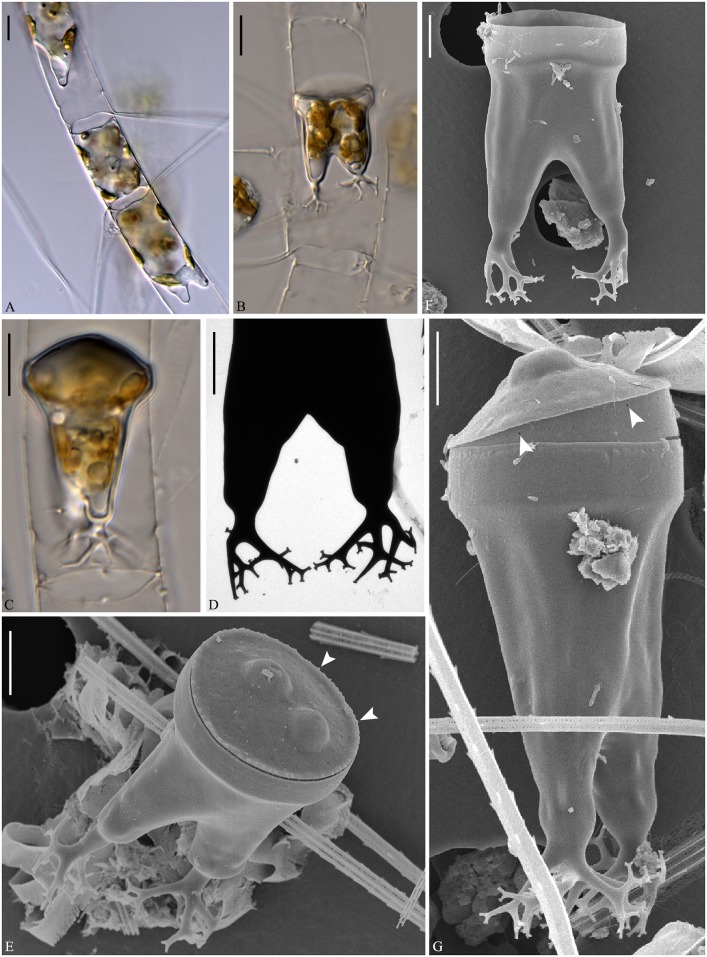
*Chaetoceros mitra* resting spores. LM (A–C), TEM (D) and SEM (E–G); strain P10A1. A: Early stage of resting spore formation. B and C: Mature resting spores within mother cells. D: Two elongated processes with dichotomous branches distally. E–G: Resting spores in different views, showing a row of silica warts along the secondary valve edge (arrowheads in E) and a ring of puncta at the secondary valve mantle (arrowheads in G). A–C scale bars, 10 μm. D–G scale bars, 5 μm.

***Chaetoceros mitra*** (Bailey) Cleve 1896 p.4, Pl I, fig 6 emend. Li, Boonprakob, Moestrup & Lundholm Figs 13–15 and 20A–20C

Basionym: *Dicladia mitra* Bailey 1856

Synonym: *D*. *groenlandica* Cleve 1873

Lectotype designated here: fig 2, plate II in Cleve [18] (shown as Fig 20C). A holotype does not exist and a lectotype has therefore been selected.

Epitype designated here: Fixed material of strain P10A1, from Tromsø, Norway. Material has been deposited at the Natural History Museum of Denmark, Copenhagen (C-A-92072). Figs 13–15 illustrate the epitype. Sequences of D1-D3 LSU rDNA (Genbank accession number KX065247) and SSU rDNA (KX611427) represent the epitype.

Type locality: Sea of Kamtschatka

Chains are straight (Fig 13A), or slightly curved (not shown). Several chloroplasts are present in each cell (often 6–14). In broad girdle view, cells are usually rectangular, the pervalvar axis often longer than the apical axis (Fig 13A and 13B). In valve view, the valves are broadly elliptical to round-oval (Figs 13C, 14G and 14H). The valve face is saddle-shaped, as the central region of the valve face is slightly raised (Figs 13C and 14G). The valve face edges are broadly arc shaped and marked by an elevated silica rib (Figs 13C and 14G). On the valve surface, costae diverge from a central annulus (Fig 14H), without distinct poroids between the costae. A constriction is located at the border between the mantle and the girdle bands (Fig 13B, arrows). The mantle occupies one third to one fifth of the pervalvar axis, but sometimes less–as little as one tenth during resting spore formation (Fig 15A and 15C). The mantle is ornamented with narrow parallel rows of costae (Fig 14G). A furrow is situated above the basal ring of the mantle (Fig 14F and 14G, arrows). Apertures are narrow oval to hexagonal, sometimes slightly indented in the middle (Fig 13A and 13B).

The setae of a chain are diverge from the apical plane (Brunel group II) (Fig 13A). Setae are soft and more or less curved and protrude from the corners of the cell (Fig 13A and 13B). The terminal setae have almost the same orientation as the intercalary setae (Fig 13A–13C) or they are slightly V-shaped in broad girdle view. Sibling setae cross over at the chain border, with no basal parts (Figs 13A, 13B and 14F). Silicified wing-like structures are present near the seta base and form a bridge between sibling cells (Fig 14F, arrowhead). They also form a continuation of the silica rib along the valve face edge on the intercalary valves. On the terminal valve they are replaced by fringes (Fig 14G, curved arrow). The setae are four-six sided with four to six rows of poroids and spines arranged alternatingly on the setae (Fig 14B–14E). Poroids of the setae are round-oval (Fig 14B–14E), 0.2±0.1 μm in size, 39.8±7.4 poroids in 10 μm (n>20), the density varying within a single seta. The poroids are barely visible in LM (Fig 14A)(Table 1). Poroids near the seta bases are slightly smaller and more scattered (Fig 14F).

A single slit-like rimoportula without any external tube is situated slightly excentrically on the terminal valve (Fig 14G, arrowhead). Processes on intercalary valves are absent (Fig 14H). Several open bands are present, each with parallel costae (Fig 14I and 14J). The apical axis is 16.5–23.8 μm, the pervalvar axis 28.1–48.2 μm, the length of the aperture in the pervalvar axis 2.9–10.0 μm (n>20).

Resting spores are most often situated close to one valve of the mother cell (Fig 15A and 15C), sometimes in the middle of the cell (Fig 15B). The surface of the resting spore is mainly smooth (Fig 15E–15G). The primary valve extends into two elongated elevations with dichotomous branching processes distally (Fig 15B–15G). One or two bulges are present on the secondary valve face (Fig 15E and 15G). The elevations are 21.8–36.2 μm long, the branching processes 5.7–11.4 μm long, the apical axis 14.6–21.4 μm (Table 2). The outer slope of the elevation is almost straight (Fig 15F and 15G). Length of elevation is 2–5 times longer than the branching processes (Table 2). A single circular row of small silica warts is visible along the secondary valve edge (Fig 15E, arrowheads). The mantle touches the bands of the mother cell (Fig 15A–15C). A ring of puncta is present at the margin of secondary valve mantle (Fig 15G, arrowheads).

Geographical distribution: Greenland (April, present study); Sea of Kamtschatka [17]; Cape Wankarema, east coast of Greenland, Baffin Bay [18]; Narragansett Bay of Rhode Island [10], Gulf of St. Lawrence; Canada (as *C*. *lorenzianus* in [11]).

**Fig 16:**
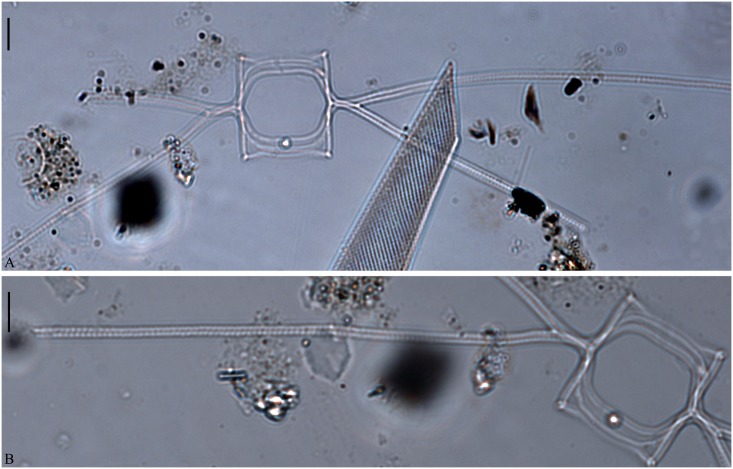
Lectotype material of *C*. *lorenzianus* from the Grunow Collection. A: Intercalary valves with oval-hexagonal aperture and some fusion of the basal parts of the setae. B: Intercalary seta poroids visible under the LM. Scale bars are 10 μm.

***C*. *lorenzianus*** Grunow 1863 p. 157, Pl 14, fig 13 Figs 16 and 20D

Lectotype designated here: A slide in a capsule (Acqu 1901/3674) with a coverslip of mica found in Grunow’s accession book under number 501 (Grunow 501). The capsule is glued onto a small paper sheet, and next to the capsule is a sketch of *C*. *lorenzianus* made by Grunow similar to fig 13 in Grunow [8] (here illustrated in Fig 20D). Fig 16A and 16B illustrate the lectotype. A holotype was not selected by Grunow.

Type locality: Adriatic Sea

Original description: Rectangular or quadrangular cells in girdle view, setae bend out, setae long, delicate with punctuation. Apical axis 20–43 μm.

Examination of the lectotype material: only a few frustules were found on the slide. (The mica was observed using a 40X objective without oil to avoid destroying it): The aperture was quadrangular-hexagonal (Fig 16A). The setae protruded from the elevated corners of the cell and were located more or less in the apical plane. Sibling setae crossed over just outside the chain border, with some fusion of the basal parts of the setae and with short basal parts present (Fig 16A and 16B). Seta poroids were large and visible in LM as punctuations (Fig 16B), with a density of 7.2±1.7 poroids in 10 μm.

### Statistics

**Fig 17:**
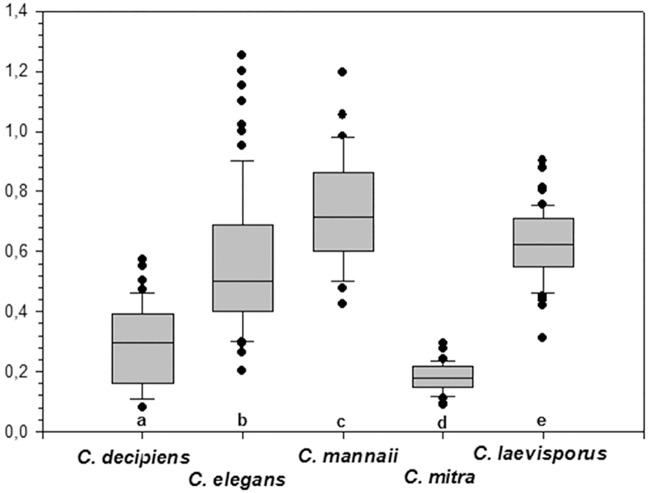
Comparison of setae poroid sizes. Small letters on the x-axis indicate statistically significant differences among taxa.

**Fig 18:**
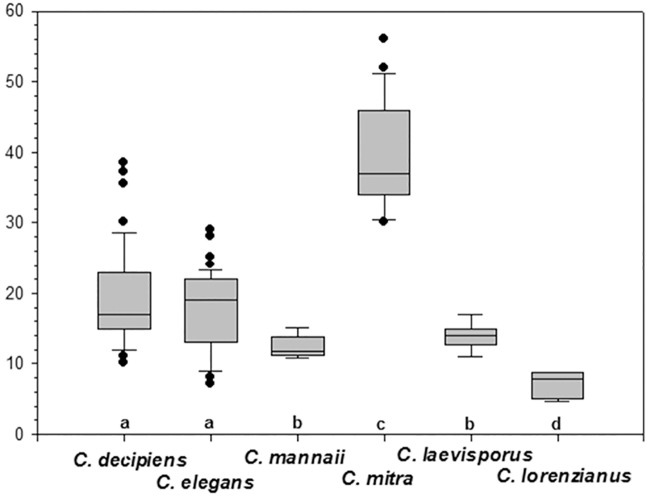
Comparison of setae poroid densities. Small letters on the x-axis indicate statistically significant differences among taxa.

One-way ANOVA analyses showed that poroid sizes on setae were significantly different among species (P<0.001) (Table 1, Fig 17). For seta poroid density, one-way ANOVA showed a clustering into four significantly different groups (P<0.0001) (Fig 18), *C*. *lorenzianus* forming a group of its own with a poroid density significantly smaller (c. 7 poroids in 10 μm) than all other taxa, and *C*. *mitra* with the highest poroid density, c. 37–40 poroids in 10 μm, forming a second group. The third group comprising *C*. *elegans* and *C*. *decipiens* with an intermediate poroid density from c. 15–23 poroids in 10 μm and a fourth group comprising *C*. *laevisporus* and *C*. *mannaii* with a an intermediate but lower poroid density of c. 12–15 poroids in 10 μm (Table 1).

In the resting spores, the apical axis, pervalvar axis of epivalve and the relationship between the length of the elevations and the branching processes were statistically different in *C*. *elegans* and *C*. *mitra* (P<0.0001) using one way ANOVA, whereas the length of the branching processes were not statistically different (P>0.05) (Table 2).

### Phylogenetic analyses

**Fig 19:**
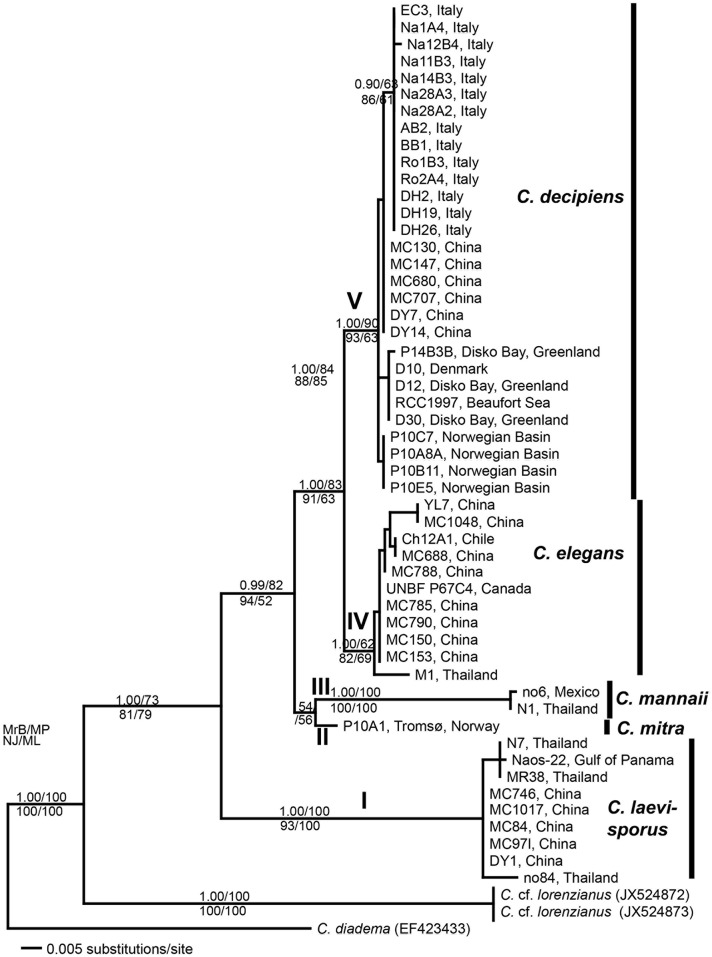
Molecular phylogenetic tree. Based on analyses of the D1-D3 region of the LSU rDNA sequences. Numbers indicated on the branches are posterior probability of Bayesian analyses (MrB) and boot strap support of neighbor joining (NJ), maximum parsimony (MP) and maximum likelihood (ML) analyses.

The MrB, NJ, MP and ML phylogenetic analyses of LSU rDNA all showed the same overall tree topology (Fig 19). A total of 54 ingroup strains of the *C*. *lorenzianus* group clustered in six well-supported clades, corresponding to the five morphotypes and an unknown separate and basal terminal clade comprising only sequences from Genbank named *C*. cf. *lorenzianus*. This clade will not be discussed further as we have no proper morphological information on the strains. The next clade to branch off (Clade I; bootstrap values = 100–93), included nine strains of *C*. *laevisporus*, all from warm water areas: three from Thailand, five from south China, and one from Gulf of Panama (EF423437, *C*. cf. *lorenzianus*). The remaining strains clustered together, supported by intermediate support (0.99/82/94/52 in MrB/MP/NJ//ML, respectively). *Chaetoceros mannaii* strains from Mexico and Thailand clustered together in a highly supported clade (1.00 or 100% in all analyses) with *C*. *mitra*, represented by one strain, as the sister group. A well-supported clade comprising eleven strains of *C*. *elegans* from Chile, south China, Thailand and Canada constituted the fourth branch (bootstrap support 1.00/62/82/69 in MrB/MP/NJ/ML, respectively). The *C*. *elegans* clade clustered together with the last also well supported clade comprising 29 strains of *C*. *decipiens* from geographically widespread localities. This clade (clade V) included three strains previously identified as *C*. *lorenzianus* [35]. The analyses of SSU rDNA support the LSU analyses, both with regard to strains of same species clustering in together and with the branching order of the clades (S3 Fig, S2 Table).

Within the *C*. *decipiens* clade, the strains clustered into four main groups differently supported in the LSU analyses. The Italian strains clustered separately in most analyses, and often close to the Chinese strains. The strains from colder waters tended to cluster separately from the others, some of them in a well-supported clade comprising strains from Denmark, Greenland, and Beaufort Sea, the others in a clade comprising strains from the Norwegian Sea only. Seven base pair positions out of a total of 748 varied among the *C*. *decipiens* strains. The Italian strains differed from all other strains in two positions, one position separated the cold-water strains from the remaining strains, and the rest of the variation was observed among the cold-water strains, which were genetically most diverse.

## Discussion

The morphological and molecular analyses showed a higher species diversity of the *C*. *lorenzianus* complex than presently known.

The group itself was found to be characterized by the following morphological features: 1) straight chains with stiff setae, 2) several chloroplasts in each cell (four to more than ten), 3) square or rectangular cells in broad girdle view, 4) saddle-shaped valves, 5) oval to hexagonal apertures, 6) four-six sided setae with longitudinal rows of poroids and spines, 7) terminal setae differing more or less in direction from the intercalary setae, and 8) silica ears or fringes present at the base of the setae.

At the species level, characters found to be useful for identification were (Table 1): 1) shape, size and density of the seta poroids (Figs 17 and 18), 2) orientation of the setae, 3) shape and size of the apertures, 4) presence or absence of basal parts on the setae, 5) presence or absence of ornamentations on the valve face and mantle, and 6) the structure of the resting spore. The size, shape and density of the seta poroids we found to be our first choice of character when identifying species in this group, supporting the recent suggestion by [37, 38] to extend the use of morphological characters to include characters of the setae. Poroid size differed among all the species although with some overlap (Fig 17). Poroid shape was unique for *C*. *elegans*, and poroid density grouped the species in four significantly different groups (Fig 18).

When the present study began, section *Dicladia* comprised only three species. Following the finding of three additional species, and because we are aware of additional undescribed species, it becomes necessary to carefully consider problems associated with identification of the individual species. This will be discussed under each species below. For all species, a combination of characters is needed for reliable identification.

### *Chaetoceros* *mitra*

**Fig 20:**
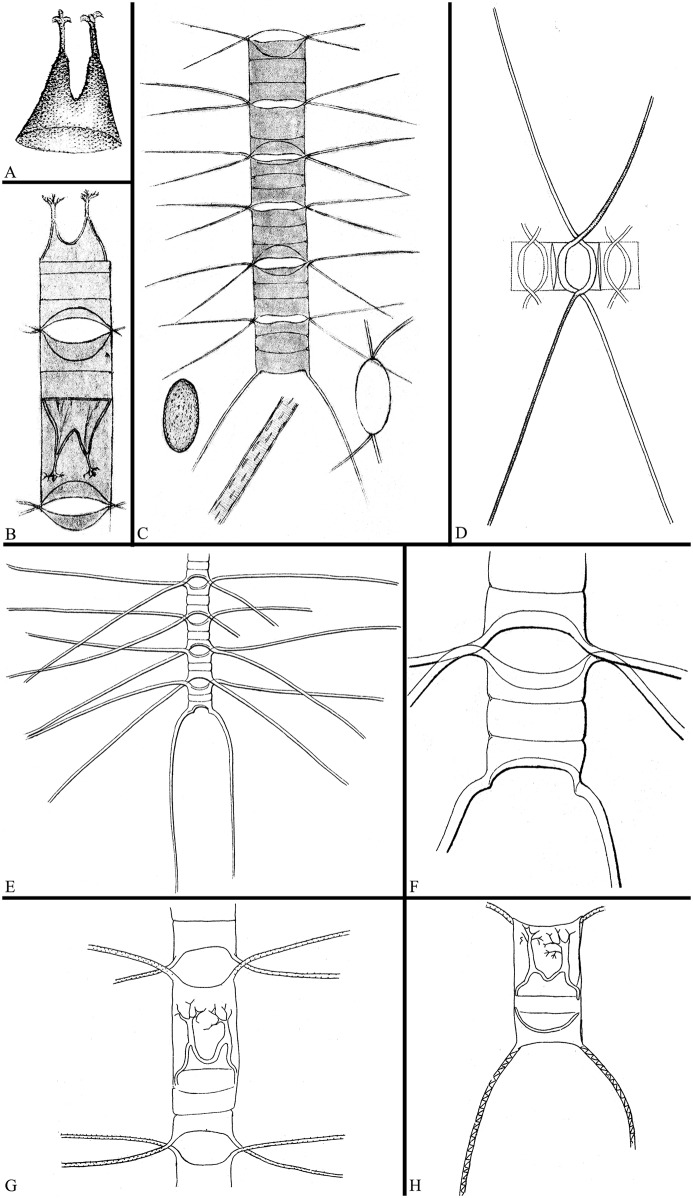
The original drawings of species of the *C*. *lorenzianus* complex. A: Resting spore of *C*. *mitra* by Bailey [17]. B and C: *C*. *mitra* by Cleve [18]. Vegetative chain, intercalary valve, terminal seta and apical view of seta positions (C), and chain with resting spores (B, lectotype). D: Vegetative chain of *C*. *lorenzianus* by Grunow [8]. E and F: *Chaetoceros decipiens* by Cleve [7]. G and H: Resting spores of *C*. *lorenzianus* by Okamura [39].

This was the first species of the *C*. *lorenzianus* complex to be described, as *Dicladia mitra* from the Sea of Kamtschatka (Fig 20A) [17] based only on a valve of a resting spore. When chains of cells were found, the taxon was transferred to *Chaetoceros* [18]. Cleve described the cells as forming straight chains with narrow peanut-shaped to narrow elliptical apertures, concave valves and strong terminal setae diverging c. 90°, and diverging from the apical plane (Brunel Group II) with spirally arranged puncta and indistinct transverse striations (Fig 20B and 20C) [18]. This agrees with our observations, except for the spiral arrangement of puncta. Cleve’s drawings (loc. cit. Fig 1C) show a pattern of spines or puncta on the spines similar to what we have seen (Fig 14A–14D), perhaps spirally inserted spines.

*Chaetoceros mitra* can distinguished from all the other species of the complex by the sibling setae diverging 30°–80° from the apical plane, defined as Brunel Group II (Fig 13A and 13B) (Brunel 1972), as depicted by Cleve ([18], Fig 20C in the present paper). In the other species, setae are positioned more or less in the apical plane, i.e., Brunel Group I (Figs 1C–1D, 4A–4C, 8A–8B and 11A). Hasle & Syvertsen [6] stated that *C*. *mitra*, when seen in broad girdle view, has parallel or convergent terminal setae and in this way differs from *C*. *lorenzianus*, whose terminal setae are diverging. This does not agree with our findings, the terminal setae in our material of *C*. *mitra* were diverging, as illustrated by Cleve [18], Fig 20C.

The morphology of the resting spore as a distinguishing character for *C*. *mitra* needs evaluation as similar resting spores can be found in *C*. *elegans* (Fig 7A–7F) and were reported in material identified as *C*. *lorenzianus* [6, 9, 10, 39]. The differences with the *C*. *elegans* resting spore is discussed below and differentiation with the spore of *C*. *lorenzianus* must await “true” *C*. *lorenzianus* (from the type locality) being brought into culture. The illustration of a resting spore ([11], pl. 38, fig e) appears to disagree with *C*. *mitra* due to the length of the elevations, the relationship between the length of the elevation and the processes as well as the slope of the elevation. In an account of fossil *Chaetoceros* spores by [24, 40], three ‘species’ (form-species) of *Dicladia* are treated. Suto’s *Dicladia mitra* is different from our Fig 15B of *Chaetoceros mitra*. The relationship between the recent and the fossil taxa is unknown.

The size and density of the seta poroids (Fig 14B–14E) differentiates *C*. *mitra* from all the other species in the complex (Table 1), as *C*. *mitra* has the smallest type of seta poroids (Fig 17), measuring 0.2 ± 0.1 um, a size that can hardly be resolved in LM. The shape of the apertures is similar in *C*. *decipiens* and *C*. *mitra*, and poroid sizes overlap, but the two species *Chaetoceros decipiens* and *C*. *mitra* differ from each other in the density of setae poroids, presence/absence of fusion of the seta bases, the divergence of the intercalary setae from the apical plane (compare Figs 1D and 13B) and the ornamentation of the valves. While *C*. *decipiens* has scattered poroids, *C*. *mitra* lacks poroids on the valve face (compare Figs 2H and 14H).

### *Chaetoceros decipiens* and *C*. *lorenzianus*

The problems of identifying species in section *Dicladia* have focused mainly on *C*. *decipiens* and *C*. *lorenzianus* being morphologically similar with overlapping distribution.

Nine of our strains of *C*. *decipiens* originated from Greenland, Denmark Strait, Norwegian Sea and Denmark, overlapping with the original localities of *C*. *decipiens* in the North Atlantic and the Davis Strait [7] and agreeing with the description of *C*. *decipiens* in having flat chains with small oval apertures and densely striated setae in the same plane ([7], our Fig 20E and 20F). Fusion of the sibling setae was very common in some of the chains (Fig 1C and 1D), but absent in others (Fig 1E and 1F); as in Cleve’s original illustration (Fig 20F). Fusing setae were observed both in *C*. *decipiens* and in the type material of *C*. *lorenzianus* (Fig 16A and 16B)

In the original description of *C*. *decipiens*, the striation of the setae was mentioned to be 20–25 in 25 μm, i.e. ca. 1 per μm, while fewer were present in the coarser *C*. *lorenzianus* [7]. In our cold-water material, which was otherwise typical of *C*. *decipiens*, striation of the setae was sometimes visible under LM (Fig 2A) and sometimes not (Fig 2B), and with a poroid density 19.9±6.7 in 10 μm, higher than in the original description by Cleve [7].

In *C*. *decipiens* and *C*. *elegans*, the mantle had the same ornamentation as the valve face, both being perforated by poroids (Figs 3D and 5G, respectively). Such poroids were not observed on the valve and mantle of the remaining species (Figs 8F, 9C, 12C, 12D and 14H), and these species seemed to be perforated by much smaller pores. Okuno [36] stated that the valve face of *C*. *lorenzianus* lacks distinct poroids or holes, while these are distinct in *C*. *decipiens*, a view supported by Evensen & Hasle [21].

In the present study, no strains similar to *C*. *lorenzianus* were established. Permanent slides of the type material of *C*. *lorenzianus* were obtained from Vienna and observed in the LM (Table 1). As mentioned above, coarse seta poroids have been used to distinguish *C*. *lorenzianus* from the other species [6, 7]. In the type material of *C*. *lorenzianus*, the density of setae poroids was significantly lower than in all the other species examined, but poroid size could not be established (Table 1). In material identified as *C*. *lorenzianus* by Hernández-Becerril [12] but not forming resting spores, the number of poroids in 10 μm was less than 10 (loc. cit, pl. 23, figs 3 and 4) as in the type material of *C*. *lorenzianus*. Pore size was 1.67±0.46 μm (loc. cit, pl. 23, figs 3 and 4). Similarly, material identified by Okuno [36] showed poroids which had a density of 5–7 in 10 μm and measuring around 1 μm in length (loc. cit. pl. VI, fig 7). Most other studies only used LM, and the density of setae poroids could not be ascertained. The type material of *C*. *lorenzianus* showed some fusion of the proximal parts of the setae, a character often used to differentiate *C*. *decipiens* from *C*. *lorenzianus*.

Resting spores have been considered a key character for distinguishing between *C*. *lorenzianus* and *C*. *decipiens*. Formation of resting spores could not be induced in any of our strains of *C*. *decipiens* although attempts were made with several of the strains, supporting previous observations [6, 9, 12, 13, 15, 35]. Resting spores of cf. *C*. *lorenzianus* were first reported from Japan [39] nearly fifty years after the discovery of the vegetative cells [8]. The primary valves possessed two elongated processes with dichotomous branches distally. The spores were located centrally or near one valve of elongated mother cells (our Fig 20G and 20H) [39]. Based on the morphology of the resting spores in the drawings (Fig 20G and 20H), we conclude that the spore of *C*. *lorenzianus* is probably fairly similar to that of *C*. *mitra* and *C*. *elegans*. The same type of spore has subsequently been reported by others [9, 13]. A slightly different spore type has been illustrated by drawings [6, 14, 41, 42], in which both the length of the elongated processes and the pervalvar axis of the mother cells is much shorter, which–if they represent true *C*. *lorenzianus*—would make the spore of *C*. *lorenzianus* distinct from *C*. *mitra*. Material of *C*. *lorenzianus*, preferably from the type locality, is needed to determine the morphology of the resting spore. Most studies reporting resting spores of *C*. *lorenzianus* have used LM, and details of the valves are not available. There is little doubt, however, that *C*. *lorenzianus* and *C*. *mitra* have sometimes been mixed up (S2 Table).

### Diversity of *Chaetoceros decipiens*

The phylogenetic analyses of *C*. *decipiens* included a total of 33 strains, isolated from several geographical localities. The strains clustered into four clades, more or less corresponding to the localities of origin. Not all branches were well supported in the trees, however, and inclusion of more strains from other geographical localities and sequencing of more variable genes are therefore needed to evaluate whether the existence of geographically separated clades is real. We did not observe morphological differences among the strains from the different clades, except a tendency for the cold-water strains to have s smaller poroid size than the temperate-warm-water strains.

### C*haetoceros elegans*

The species *C*. *elegans* possesses intermediate-sized poroids on the setae, 0.5 ± 0.2 μm, visible in LM. Besides the size, which is statistically different from the other examined taxa, the mostly tear-shaped setae poroids are unique (Fig 6E and 6G; Table 1). Poroid density also differentiates this species from all other taxa except *C*. *decipiens* (Table 1).

*Chaetoceros elegans* is characterized by large rounded quadrangular-rectangular apertures (Figs 4A, 4C, 5B and 5C), and differs in this respect from the other species (Table 1) except *C*. *lorenzianus*, which has been illustrated with quadrangular-hexagonal apertures (Figs 16A, 16B and 20D) [8]. A large aperture/pervalvar index also characterizes *C*. *elegans* (Table 1), and differentiates it from the other taxa except *C*. *decipiens* and *C*. *lorenzianus*. Distinct basal parts of the setae are present in *C*. *elegans* (Fig 5B and 5C). A very short basal part is present also in *C*. *mannaii* (Fig 11C and 11D) while basal parts are absent in *C*. *laevisporus*, *C*. *decipiens* and *C*. *mitra*, and apparently also in *C*. *lorenzianus* (Fig 16A and 16B). *Chaetoceros elegans* seems to be a widely distributed species. In addition to our findings in China, Thailand and Chile, sequences of specimens from Canada (previously reported as *C*. cf. *decipiens*) have been identified (Fig 19), and material which we refer to this species has been illustrated from Japan (as *C*. *decipiens* in [36]) and Gulf of California (as *C*. *lorenzianus* in [12]).

The resting spores of *C*. *elegans* and *C*. *mitra* both possess two elevations and branching processes on the primary valve and 1–2 bulges on the secondary valve. Using the terminology of Suto [24], the resting spores are distinguished from each other based on 1) the length of the apical axis 2) the length of the pervalvar axis of the primary valve, 3) the relationship between the length of the pervalvar axis of the primary valve and the length of the branching processes, 4) the slope of the elevations, and 5) the position where the elevations join (Table 2).

### *Chaetoceros* *mannaii*

Cells of *C*. *mannaii* are ultrastructurally similar to field material identified as *C*. *lorenzianus* from Thailand, Mexico and Japan ([12, 36], own observations). Compared to the original description of *C*. *lorenzianus*, *C*. *mannaii* has large poroids on the setae, 0.7±0.2 μm long, the largest among strains of section *Dicladia* examined by us, and readily visible in LM. They are more closely spaced (12.3±1.6) than in the type material of *C*. *lorenzianus* (7.2±1.7), and smaller than those of the Mexican ([12], pl. 23, figs 3 and 4) and Japanese material ([36], pl. VI, fig 7) tentatively identified as *C*. *lorenzianus*, where poroids of 1 μm or more were illustrated. In addition, the apertures of *C*. *mannaii* are hexagonal, compared to the more quadrangular apertures of *C*. *lorenzianus* (compare Figs 11C, 11d, 16A and 16B) [8]. *Chaetoceros mannaii* also differs in having a smaller apical axis, 5.7–12.9 μm, compared to 20–43 μm in *C*. *lorenzianus* [8], 13–25 μm in the Mexican material [12] and around 30 μm (with a variation of 10–80 μm) in the Japanese material [36].

Cells of *C*. *mannaii* are otherwise characterized by heavily silicified frustules and a distinct furrow above the basal ring of the mantle (Fig 11F, arrowheads). The terminal cell of *C*. *mannaii* carries a relatively long and distinct external tube of the rimoportula (Figs 11E, 11F and 12C). An external tube was not observed in *C*. *decipiens*, *C*. *laevisporus* and *C*. *mitra* (Figs 2F, 8E and 14G), and a very short one was present in *C*. *elegans* (Fig 5D and 5E). This character still needs to be explored in *C*. *lorenzianus*.

Resting spores were not observed in *C*. *mannaii* although several attempts were made to induce spore formation.

### *Chaetoceros* *laevisporus*

This is the only species in the *C*. *lorenzianus* complex with smooth resting spores without processes, and in the phylogenetic tree the strains form a separate branch. The smooth resting spore (Fig 10A and 10B) differs markedly from the spores of *C*. *lorenzianus*, *C*. *mitra* and *C*. *elegans* with their dichotomously-branching extensions. Furthermore the apertures are oval-peanut shaped in *C*. *laevisporus* (Figs 8A, 8B, 8D and 10A), thus differing from most other taxa in the complex. Seta poroid density is intermediate, 13.8±1.9 in 10 μm as in *C*. *mannaii* (Table 1), but different from all the other taxa. Poroid size on the setae was assessed to be c. 0.6 μm, slightly less than in *C*. *mannaii*, but the poroids were still visible in LM (Fig 8C).

### Additional morphological characters in section Dicladia?

A feature of *C*. *laevisporus* which occurs also in *C*. *decipiens* and *C*. *elegans* (Figs 1G and 4D, arrowheads) is the presence of a non-silicified, V-shaped protrusion on the terminal valves (Fig 8A, arrowhead). *Chaetoceros* cf. *lorenzianus* from the Gulf of Panama shows the same feature [35]. This, together with the reduced external tube of rimoportula on the terminal valve (loc. cit. figs 77–80 in [35]) clearly places this material as belonging to *C*. *laevisporus*, The V-structure was observed in *C*. *decipiens* by Rines & Hargraves [10] and Jensen & Moestrup [9], and may be present in more species of the section. Because of its non-silicified nature, the structure disappears in acid-cleaned material.

The ear-like structures at the marginal border of the apertures have been observed in all the studied strains, often in different shapes on the intercalary valves and terminal valves. In *C*. *decipiens* and *C*. *mitra*, the structures are very distinct, and they interconnect the edges of sibling valves on each side of the aperture (Figs 3B, 3C and 14F). In *C*. *elegans*, *C*. *laevisporus* and *C*. *mannaii* they are smaller, sometimes with overlapping parts (Figs 6A, 6B, 9A, 9B, 11C and 11D). On the terminal valves, these structures vary in shape, as fringes in *C*. *decipiens*, *C*. *laevisporus* and *C*. *mitra* (Figs 2F, 9C and 14G) and as slice-shaped structures in *C*. *elegans* and *C*. *mannaii* (Figs 5D and 11E). Based on the morphological variation observed, and the potential influence of environmental factors on the development of the structures, it is somewhat uncertain whether they can be used as a species-specific character.

Although our study was based on established strains, with the risk of culture condition artefacts affecting the morphology, we regard the present species delineation sound, due to the following: 1) strains were fixed soon after establishment and morphology is thus not very likely affected by artefacts, 2) several of the species are based on strains from separate locations showing the same morphology, 3) observations of field samples made it possible for us to identify the taxa based on the descriptions provided, 4) evaluations with previously published records of species belonging to *Dicladia*, for which electron micrographs are available (S3 Table) support our observations and 5) the study was performed over a long period, and subsequently established strains confirmed the species descriptions.

## Conclusions

When the present study was begun, three species were known in section *Dicladia*, two of which could not be distinguished from each other with certainty. Our studies have shown that the group is distributed worldwide, and six species have now been found, which according to our information are geographically widely distributed.

*C*. *decipiens* occurs worldwide, from the Arctic to the tropics. *C*. *mitra* is the only cold-water species, reported from the Arctic and adjacent cold waters. *C*. *elegans*, *C*. *laevisporus* and *C*. *mannaii* all appear to be warm-temperate water species. *C*. *lorenzianus* has been reported globally, but we have found material only from the type locality in the Adriatic Sea, other reports do not appear to agree with this material. We hope that the observations presented in the present report will stimulate further studies on this species complex, including “true” *C*. *lorenzianus*, and provide information, not only on the molecular phylogeny of this elusive species, but also on its geographical distribution.

## Supporting Information

S1 Fig Auxospores of *Chaetoceros decipiens* under LM; strain Ro2A4. Fig 1: Three auxospores (arrows) forming on the girdle of the mother cells. Scale bar 20 μm.
(TIF)

S2 Fig Auxospore of *Chaetoceros decipiens* under LM; strain Ro2A4. Fig 2: A nearly developed daughter colony and an auxospore (arrow). Scale bar 20 μm.
(TIF)

S3 Fig Molecular phylogenetic tree based on analyses of the SSU rDNA sequences. Numbers indicated on the branches are posterior probability of Bayesian analyses (MrB) and bootstrap support of neighbor joining (NJ), maximum parsimony (MP) and maximum likelihood (ML) analyses.
(TIF)

S1 Table List of cultures of the *C*. *lorenzianus* complex used in molecular analysis inferred from LSU, showing strain designation, sampling location and date, as well as LSU accession number. NA means no molecular data.
(DOCX)

S2 Table List of cultures of the *C*. *lorenzianus* complex used in molecular analysis inferred from SSU, showing strain designation, sampling location and date, as well as SSU accession number. (DOCX)

S3 Table Previous records of species in the *C*. *lorenzianus* complex. Evaluation of previously published records of species in the *C*. *lorenzianus* complex using EM.
(DOCX)

## References

[1] Mann DG, Droop SJM. Biodiversity, biogeography and conservation of diatoms. Hydrobiol. 1996;336: 19–32

[2] Mann DG, Vanormelingen P. An inordinate fondness? The number, distributions and origins of diatom species. J Eukar Microbiol. 2013;60: 414–420 10.1111/jeu.12047 23710621

[3] Kooistra WHCF, Sarno D, Balzano S, Gu H, Andersen RA, Zingone A. Global diversity and biogeography of *Skeletonema* species (Bacillariophyta). Protist. 2008;159: 177–193 10.1016/j.protis.2007.09.004 18042429

[4] Lundholm N, Bates SS, Baugh KA, Bill BD, Connell LB, Léger C et al Cryptic and pseudo-cryptic diversity in diatoms—with descriptions of *Pseudo-nitzschia hasleana* sp. nov. and *P*. *fryxelliana* sp. nov. J Phycol. 2012;48: 436–454 10.1111/j.1529-8817.2012.01132.x 27009733

[5] Guiry MD & Guiry GM. Algaebase. World-wide electronic publication, National University of Ireland, Galway http://www.algaebase.org (last update 1-April 2016)

[6] Hasle GR, Syvertsen EE. Marine Diatoms In Tomas CR (ed) Identifying Marine Phytoplankton. Academic Press, London;1997 pp 5–387

[7] Cleve PT. On diatoms from the Arctic Sea. Bihang till K Svenska Vet-Akad Handlingar. 1873;1: 1–28

[8] Grunow A. Über einige neue und ungenügend bekannte Arten und Gattungen von Diatomaceen. Verhandl kaiserl-königl zool-bot Ges. 1863;13: 137–162

[9] Jensen KG, Moestrup Ø. The genus *Chaetoceros* (Bacillariophyceae) in inner Danish coastal waters. Opera Bot. 1998;133: 5–68

[10] Rines JEB, Hargraves PE. The *Chaetoceros* Ehrenberg (Bacillariophyceae) flora of Narragansett Bay, Rhode Island, USA. Bibl Phycol. 1988; 79: 5–196

[11] Berard-Therriault L, Poulin M, Bosse M. Guide d’identification du phytoplancton marin de l’estuaire et du golfe du Saint-Laurent incluant également certains protozoaires. Publ. spec. can. sci. halieut. aquat. 1999; 128: 1–387.

[12] Hernández-Becerril DU. A morphological study of *Chaetoceros* species (Bacillariophyta) from the plankton of the Pacific Ocean of Mexico. Bull Nat Hist Mus Lond (Bot). 1996; 26: 1–73

[13] Shevchenko OG, Orlova TYu, Hernández-Becerril DU. The genus *Chaetoceros* (Bacillariophyta) from Peter the Great Bay, Sea of Japan. Bot Mar. 2006;49: 236–258

[14] Guo YJ, Qian SB. Flora Algarum Marinarum Sinicarum Tomus V. Bacillariophyta, No.1 Centricae. Beijing: Science Press; 2003 pp 345–346

[15] Hernández-Becerril DU, Flores Granados C. Species of the diatom genus *Chaetoceros* (Bacillariophyceae) in the plankton from the Southern Gulf of Mexico. Bot Mar. 1998; 41: 505–519

[16] Wang Y, Nie R, Li Y, Lu SH. Species diversity and geographical distribution of *Chaetoceros* in Guangdong coastal waters. Adv Mar Sci. 2010;28: 342–352

[17] Bailey JW. Notice of microscopic forms found in the soundings of the Sea of Kamtschatka. Am J Sci Arts Sec Ser. 1856;22: 1–6

[18] Cleve PT. Diatoms from Baffins Bay and Davis Strait. K Svenska Vet-Akad Handl. 1896;22: 3–22

[19] Throndsen J, Hasle GR, Tangen K. Norsk Kystplankton Flora. Almater Forlag AS, Oslo 2003.

[20] Cupp E. Marine plankton diatoms of the west coast of North America. Bull Scripps Inst Oceanogr. 1943;5: 1–237

[21] Evensen DL, Hasle GR. The morphology of some *Chaetoceros* (Bacillariophyceae) species as seen in the electron microscopes. Nova Hedw, Beih. 1975;53: 152–174

[22] Guillard RRL, Hargraves PE. *Stichochrysis immobilis* is a diatom, not a chrysophyte. Phycologia 1993;32: 234–236

[23] Kraus D (2014) Daniel’s XL Toolbox addin for Excel, version 6.10. 2014. http://xltoolbox.sourceforge.net.

[24] Suto I. Taxonomy of the marine resting spore genera *Dicladia* Ehrenberg, *Monocladia* gen. nov. and *Synendrum* Ehrenberg and their stratigraphic significance in Miocene strata. Diatom Res. 2003;18: 331–356.

[25] Lundholm N, Daugbjerg N, Moestrup Ø. Phylogeny of the Bacillariaceae with emphasis on the genus *Pseudo-nitzschia* (Bacillariophyceae) based on partial LSU rDNA. Eur J Phycol. 2002;37: 115–134

[26] Scholin CA, Herzog M, Sogin M, Anderson DM. Identification of group- and strain-specific genetic markers for globally distributed *Alexandrium* (Dinophyceae). II. Sequence analysis of a fragment of the LSU rDNA. J Phycol. 1994;30: 999–1011

[27] Nunn GB, Theisen BF, Christensen B, Arctander P. Simplicity-correlated size growth of the nuclear 28S ribosomal RNA D3 expansion segment in the crustacean order Isopoda. J Mol Evol. 1996;42: 211–223 8919873 10.1007/BF02198847

[28] Zhen Y, Mi TZ, Yu ZG. Detection of *Phaeocystis globosa* using sandwich hybridization integrated with nuclease protection assay (NPA-SH). J Environ Sci, 2008;20: 1481–1486 10.1016/s1001-0742(08)62553-x 19209636

[29] Alverson AJ, Jansen RK, Theriot EC. Bridging the Rubicon: Phylogenetic analysis reveals repeated colonizations of marine and fresh waters by thalassiosiroid diatoms. Mol Phylogenet Evol. 2007;45: 193–210 10.1016/j.ympev.2007.03.024 17553708

[30] Hall TA. BioEdit: a user-friendly biological sequence alignment editor and analysis program for window 95/98/NT. Nucleic Acids Symp. 1999;41: 95–98

[31] Swofford DL. PAUP*: Phylogenetic analysis using parsimony (* and other methods), Vers. 4 Sinauer Ass., Sunderland, Mass., USA 2003

[32] Posada D, Crandall KA. Modeltest: testing the model of DNA substitution. Bioinformatics. 1998;14: 817–818 9918953 10.1093/bioinformatics/14.9.817

[33] Ronquist F, Huelsenbeck JP. MRBAYES 3: Bayesian phylogenetic inference under mixed models. Bioinformatics. 2003; 19: 1572–1574 12912839 10.1093/bioinformatics/btg180

[34] Brunel J. Orientation of setae in the genus *Chaetoceros*, in regard to the apical axis. J Mar Biol Ass India. 1972;14: 315–327

[35] Kooistra WHCF, Sarno D, Hernandez-Becerril DU, Assmy P, Prisco CD, Montresor M. Comparative molecular and morphological phylogenetic analyses of taxa in the Chaetocerataceae (Bacillariophyta). Phycologia. 2010;49: 471–500

[36] Okuno H. Electron microscopical study on fine structure of diatom frustules. XIV. Observation on the genus *Chaetoceros*. Bot Mag, Tokyo. 1956;69: 182–200

[37] Lee SD, Joo HM & Lee JH. Critical criteria for identification of the genus *Chaetoceros* (Bacillariophyceae) based on setae ultrastructure. II. Subgenus *Hyalochaete*. Phycologia. 2014;53: 614–638

[38] Lee SD, Park JS, Yun SM & Lee JH. Critical criteria for identification of the genus *Chaetoceros* (Bacillariophyceae) based on setae ultrastructure. I. Subgenus *Chaetoceros*. Phycologia. 2014;53: 174–187

[39] Okamura K. Some littoral diatoms of Japan. Rep Imper Fish Inst Tokyo Japan. 1911;7: 3–18

[40] Suto I. Taxonomy and biostratigraphy of the fossil marine diatom resting spore genera *Dicladia* Ehrenberg, *Monocladia* Suto and *Syndendrium* Ehrenberg in the North Pacific and Norwegian Sea. Diatom Res. 2005;20: 351–374.

[41] Chin TG. A list of Chinese diatoms from 1847 to 1946. Amoy Fish Bull. 1951;1: 145–230

[42] Chin TG, Chen JH, Huang KG, eds. Marine Planktonic Diatoms from China Sea. Shanghai Science and Technology Press, Shanghai, 116 p. 1965.

