# Supplementary figures and images for: Diversity in the Globally Distributed Diatom Genus Chaetoceros (Bacillariophyceae): Three New Species from Warm-Temperate Waters
Source: PLoS One. 2017 Jan 13;12(1):e0168887. doi: 10.1371/journal.pone.0168887 (PMC5235366; doi:10.1371/journal.pone.0168887)

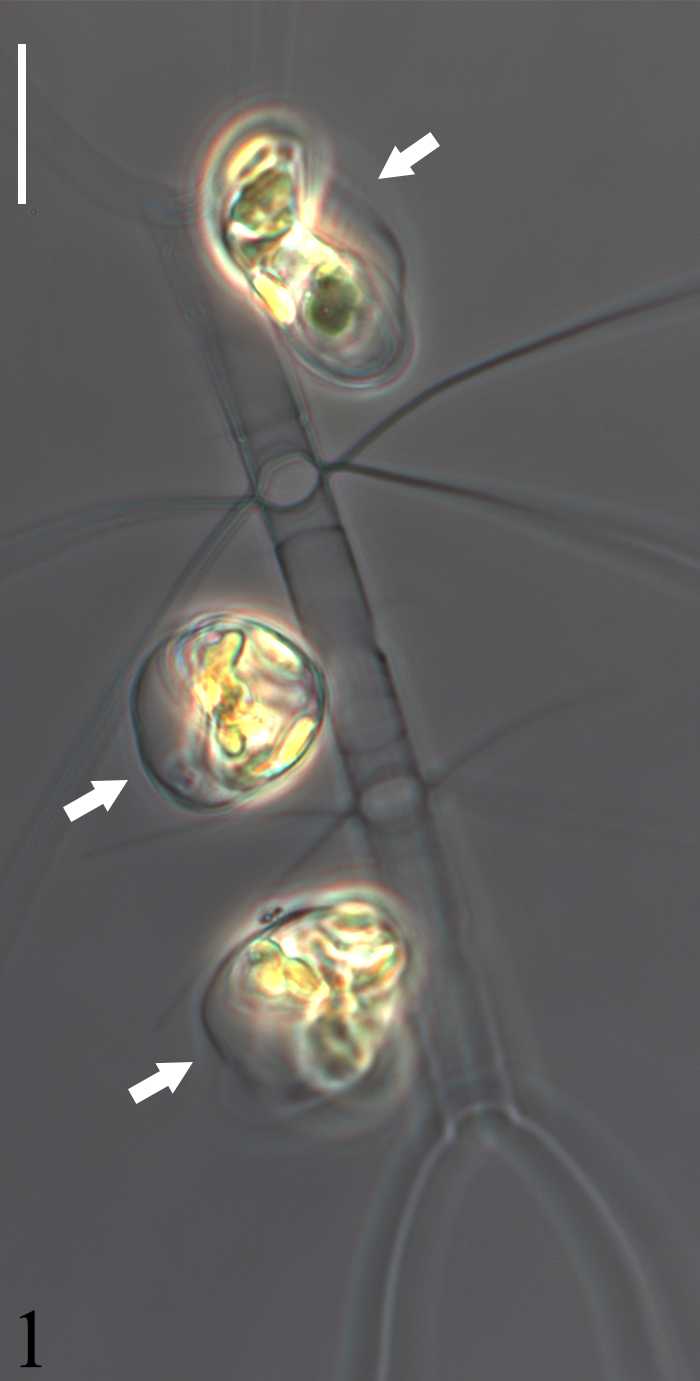

Supplement: S1 Fig — Fig 1: Three auxospores (arrows) forming on the girdle of the mother cells. Scale bar 20 μm. (TIF) [file pone.0168887.s001.tif]

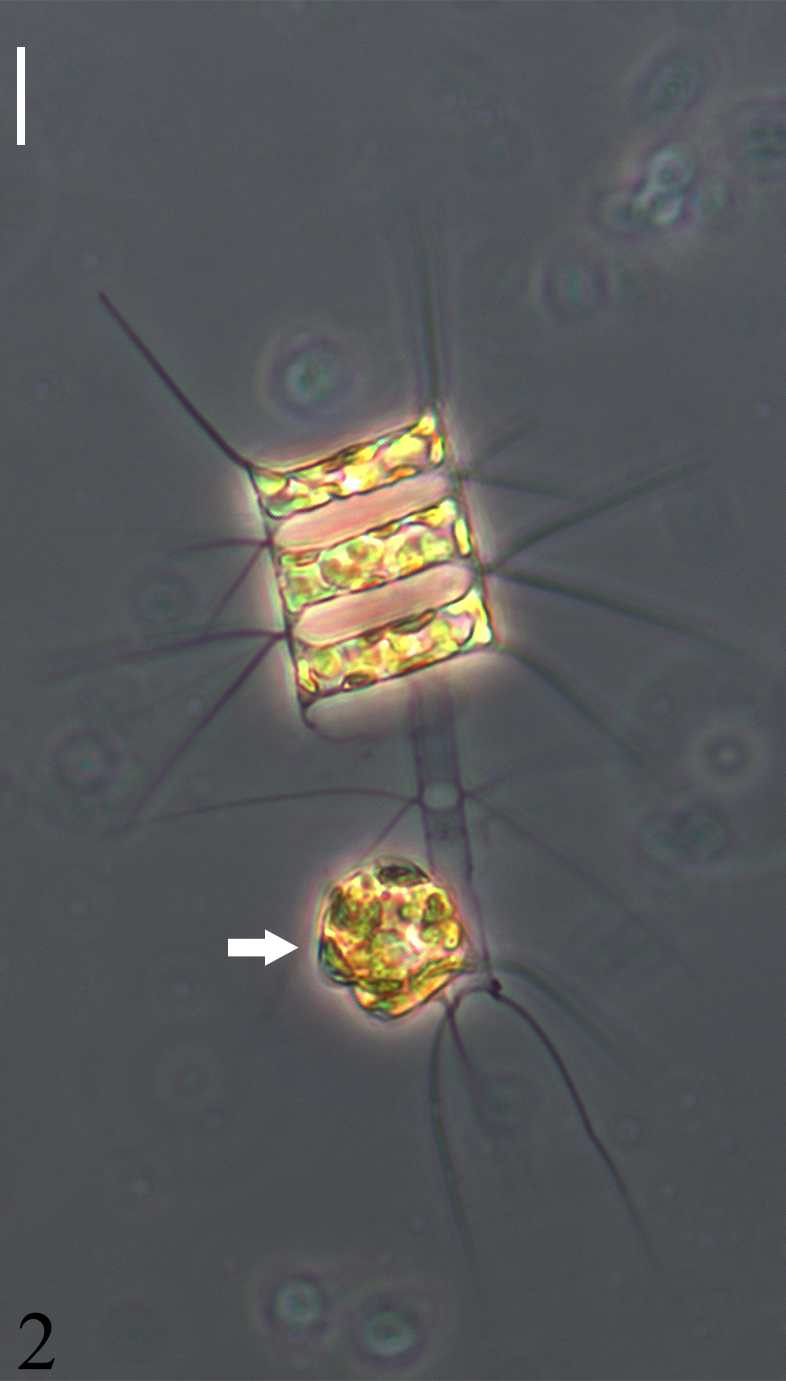

Supplement: S2 Fig — Fig 2: A nearly developed daughter colony and an auxospore (arrow). Scale bar 20 μm. (TIF) [file pone.0168887.s002.tif]

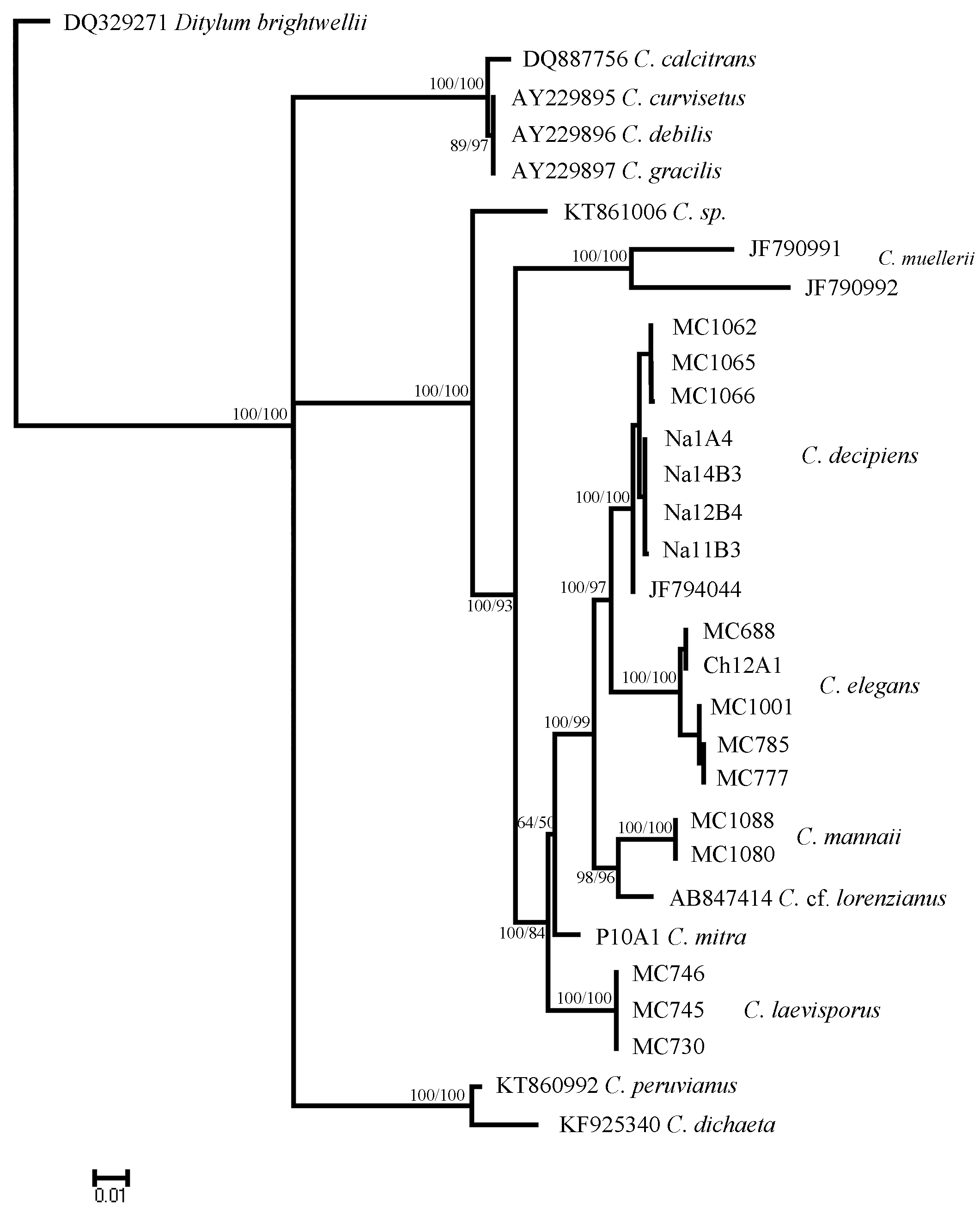

Supplement: S3 Fig — Numbers indicated on the branches are posterior probability of Bayesian analyses (MrB) and bootstrap support of neighbor joining (NJ), maximum parsimony (MP) and maximum likelihood (ML) analyses. (TIF) [file pone.0168887.s003.tif]
