# Supplementary material for: Diversity in the Globally Distributed Diatom Genus Chaetoceros (Bacillariophyceae): Three New Species from Warm-Temperate Waters
Source: PLoS One. 2017 Jan 13;12(1):e0168887. doi: 10.1371/journal.pone.0168887 (PMC5235366; doi:10.1371/journal.pone.0168887)
Supplement: S1 Table — NA means no molecular data. (DOCX) [file pone.0168887.s004.docx]

**Table S1.** List of cultures of the *C. lorenzianus* complex used in molecular analysis inferred from LSU, showing strain designation, sampling location and date, as well as LSU accession number. NA means no molecular data.

| **Species** | **Strain code** | **Date** | **Site collection** | **LSU Accession number** |
| --- | --- | --- | --- | --- |
| *C. decipiens* | D10 | Apr. 7, 2010 | Skovshoved Harbour, The Sound, Denmark, 55.75 N, 12.5833 W | KX065248 |
|  | P14B3B | June 1, 2010 | Denmark Strait, 66.0991 N, 27.5158 W | KX065218 |
|  | D12 | Apr. 26, 2011 | Disko Bay, Greenland 69.2783 N, 53.4352 W | KX065219 |
|  | D30 | Apr. 26, 2011 | Disko Bay, Greenland 69.2333 N, 53.40 W | KX065220 |
|  | P10B11 | May 30, 2010 | Norwegian Sea 67.9050 N, 4.3238 W | KX065221 |
|  | P10C7 | May 30, 2010 | Norwegian Sea, 67.9050 N, 4.3238 W | KX065222 |
|  | P10E5 | May 30, 2010 | Norwegian Sea, 67.9050 N, 4.3238 W | KX065223 |
|  | P10A8A | May 30, 2010 | Norwegian Sea, 67.9050 N, 4.3238 W | KX065224 |
|  | DH2 | May 2004 | Gulf of Naples, LTER-MC, Italy, 40.8093 N, 14.2498 E | EF423434 |
|  | DH19 | Jun. 2004 | Gulf of Naples, LTER-MC, Italy, 40.8093 N, 14.2498 E | EF423435 |
|  | DH26 | Jul. 2004 | Gulf of Naples, LTER-MC, Italy, 40.8093 N, 14.2498 E | EF423436 |
|  | EC3 | April 06, 2013 | Gulf of Naples, LTER-MC, Italy, 40.8093 N, 14.2498 E | KY129892 |
|  | AB2 | April 06, 2013 | Gulf of Naples, LTER-MC, Italy, 40.8093 N, 14.2498 E | KY129893 |
|  | BB1 | April 06, 2013 | Gulf of Naples, LTER-MC, Italy, 40.8093 N, 14.2498 E | KY129894 |
|  | MC130 | Feb 19, 2013 | Daya Bay, China, 22.7214 N, 114.5375 E | KX065225 |
|  | MC147 | June 1, 2013 | Guishan Island, China, 22.0475 N, 113.4184 E | KX065226 |
|  | MC680 | Mar 24, 2015 | Daya Bay, China, 22.7222 N, 114.5383 E | KX065227 |
|  | MC707 | May 13, 2015 | Weizhou Island, China, 21.0506 N, 109.1228 E | KX065228 |
|  | RCC1997 | Jul. 2009 | Beaufort Sea, [69.49 N 137.99 W](http://www.ncbi.nlm.nih.gov/projects/Sequin/latlonview.html?lat=69.49&lon=-137.99) | JQ995413 |
|  | DY7 | Dec. 16, 2010 | Daya Bay, China, 22.7214 N, 114.5375 E | KX065229 |
|  | DY14 | Dec. 16, 2010 | Daya Bay, China, 22.7214 N, 114.5375 E | KX065230 |
|  | Na11B3 | Mar. 19, 2014 | Gulf of Naples, LTER-MC, Italy, 40.8093 N, 14.2498 E | KY129895 |
|  | Na12B4 | Mar. 19, 2014 | Gulf of Naples, LTER-MC, Italy, 40.8093 N, 14.2498 E | KY129896 |
|  | Na14B3 | Mar. 19, 2014 | Gulf of Naples, LTER-MC, Italy, 40.8093 N, 14.2498 E | KY129897 |
|  | Na18B4 | July 1, 2014 | Gulf of Naples, LTER-MC, Italy, 40.8093 N, 14.2498 E | NA |
|  | Na1A4 | Nov. 26, 2013 | Gulf of Naples, LTER-MC, Italy, 40.8093 N, 14.2498 E | KY129898 |
|  | Na28A2 | Oct. 7, 2014 | Gulf of Naples, LTER-MC, Italy, 40.8093 N, 14.2498 E | KY129899 |
|  | Na28A3 | Oct. 7, 2014 | Gulf of Naples, LTER-MC, Italy, 40.8093 N, 14.2498 E | KY129900 |
|  | Na2B4 | Nov. 26, 2013 | Gulf of Naples, LTER-MC, Italy, 40.8093 N, 14.2498 E | NA |
|  | Na33A3 | July 14, 2015 | Gulf of Naples, LTER-MC, Italy, 40.8093 N, 14.2498 E | NA |
|  | Na33B4 | July 14, 2015 | Gulf of Naples, LTER-MC, Italy, 40.8093 N, 14.2498 E | NA |
|  | Ro1B3 | Aug. 11, 2014 | Roscoff, France, 48.7335 N, 03.9833 W | KY129901 |
|  | Ro1C2 | Aug. 11, 2014 | Roscoff, France, 48.7335 N, 03.9833 W | NA |
|  | Ro2A4 | Aug. 11, 2014 | Roscoff, France, 48.7335 N, 03.9833 W | KY129902 |
|  | Ro2C4 | Aug. 11, 2014 | Roscoff, France, 48.7335 N, 03.9833 W | NA |
| *C. elegans* | M1 | June 2, 2008 | Mannai Island, Rayong Province, Thailand, 12.6111 N, 101.6836 E | KX065231 |
|  | YL7 | Aug. 27, 010 | Dapeng Bay, China, 22.5933 N, 114.3991 E | KX065232 |
|  | MC1048 | Sep 30, 2015 | Dapeng Bay, China, 22.5953 N, 114.3981 E | KX065233 |
|  | MC688 | Apr 3, 2015 | Victoria Harbour, Hong Kong, China, 22.2960 N, 114.1840 E | KX065234 |
|  | UNBF P67C4 | Sep. 7, 2010 | New Brunswick, Canada, 45.00 N 66.733 W | KC986068 |
|  | MC785 | Aug 7, 2015 | Ningbo, China, 29.8636 N,121.5611 E | KX065235 |
|  | MC788 | Aug 17, 2015 | Zhuhai, China，22.1614 N，113.3436 E | KX065236 |
|  | MC790 | Aug 17, 2015 | Zhuhai, China, 22.1614 N，113.3436 E | KX065237 |
|  | MC150 | June 1, 2013 | Guishan Island, China, 22.0472 N, 113.4175 E | KX065238 |
|  | MC153 | June 1, 2013 | Guishan Island, China, 22.0472 N, 113.4175 E | KX065239 |
|  | Ch12A1 | Oct. 29, 2013 | Concepción, Chile, 36.5133 S and 73.1291 W | KY129903 |
| *C. laevisporus* | No 84 | Dec. 10, 2010 | Loan Island, Phuket Province on Andaman Sea, Thailand, 07.9127 N, 98.0725 E | KX065250 |
|  | MR38 | Dec. 9, 2010 | Mannai Island, Rayong Province, Thailand, 12.6077 N, 101.6858 E | KX065251 |
|  | N 7 | Dec. 9, 2010 | Mannai Island, Rayong Province, Thailand, 12.6077 N, 101.6858 E | KX065240 |
|  | DY1 | Dec. 16, 2010 | Daya Bay, China, 22.7125 N, 114.5375 E | KX065241 |
|  | MC746 | July 14, 2015 | HongKong, China, 22.2960 N, 114.1840 E | KX065242 |
|  | MC1017 | Sep 9, 2015 | Jiaozhou Bay, China, 38.9180 N, 117.7556 E | KX065243 |
|  | MC84 | Mar 24, 2012 | Daya Bay, China, 22.7542 N, 114.5554 E | KX065244 |
|  | MC97 | Sep 4, 2012 | Pearl River Estuary, China, 22.7388 N, 113.6011 E | KX065245 |
|  | Naos-22 | Feb 5, 2007 | Gulf of Panama, Panama City, Panama, 08.9177 N, 79.5327 W | EF423437 |
| *C.mannaii* | N1 | Jan. 21, 2011 | Mannai Island, Rayong Province, Thailand, 12.6077 N, 101.6858 E | KX065246 |
|  | No 6 | April, 2013 | Mazatlán, Sinaloa, Mexico,  23.0736 N, 105.9486 W | KX065249 |
| *C. mitra* | P10A1 | May 27, 2010 | Tromsø Harbour, 69.6519 N 18.9533 E, Norway | KX065247 |
| *C.* cf *lorenzianus* | CHPCT1 | 2010 | Iran | JX524872 |
|  | CHPCT2 | 2010 | Iran | JX524873 |
