# Supplementary material for: Diversity in the Globally Distributed Diatom Genus Chaetoceros (Bacillariophyceae): Three New Species from Warm-Temperate Waters
Source: PLoS One. 2017 Jan 13;12(1):e0168887. doi: 10.1371/journal.pone.0168887 (PMC5235366; doi:10.1371/journal.pone.0168887)
Supplement: S2 Table — (DOCX) [file pone.0168887.s005.docx]

**Table S2.** List of cultures of the *C. lorenzianus* complex used in molecular analysis inferred from SSU, showing strain designation, sampling location and date, as well as SSU accession number.

| **Species** | **Strain code** | **Date** | **Site collection** | **SSU Accession number** |
| --- | --- | --- | --- | --- |
| *C. decipiens* | MC1062 | Oct. 31, 2015 | Wanshan Island, China, 21.5654 N,113.4342 E | KX611413 |
|  | MC1065 | Oct. 31, 2015 | Wanshan Island, China, 21.5654 N,113.4342 E | KX611414 |
|  | MC1066 | Oct. 31, 2015 | Wanshan Island, China, 21.5654 N,113.4342 E | KX611415 |
|  | Na1A4 | Nov. 26, 2013 | Gulf of Naples, LTER-MC, Italy, 40.8093 N, 14.2498 E | KX611416 |
|  | Na14B3 | Mar. 19, 2014 | Gulf of Naples, LTER-MC, Italy, 40.8093 N, 14.2498 E | KX611417 |
|  | Na12B4 | Mar. 19, 2014 | Gulf of Naples, LTER-MC, Italy, 40.8093 N, 14.2498 E | KX611418 |
|  | Na11B3 | Mar. 19, 2014 | Gulf of Naples, LTER-MC, Italy, 40.8093 N, 14.2498 E | KX611419 |
| *C. elegans* | MC688 | Apr 3, 2015 | Hong Kong, China, 22.2960 N, 114.1840 E | KX611420 |
|  | Ch12A1 | Oct. 29, 2013 | Concepción, Chile, 36.5133 S and 73.1291 W | KX611421 |
|  | MC1001 | Aug 24, 2015 | Zhanjiang, China, 21.0154 N,110.2310 E | KX611422 |
|  | MC785 | Aug 7, 2015 | Ningbo, China, 29.8636 N,121.5611 E | KX611423 |
|  | MC777 | Aug 4, 2015 | Ningbo, China, 29.8636 N,121.5611 E | KX611424 |
| *C. laevisporus* | MC746 | July 14, 2015 | HongKong, China, 22.2960 N, 114.1840 E | KX611428 |
|  | MC745 | July 14, 2015 | HongKong, China, 22.2960 N, 114.1840 E | KX611429 |
|  | MC730 | July 14, 2015 | HongKong, China, 22.2960 N, 114.1840 E | KX611430 |
| *C.mannaii* | MC1088 | Apr 3, 2016 | Taiwan Strait,China, 22.7993 N, 116.7812 E | KX611425 |
|  | MC1080 | Apr 3, 2016 | Taiwan Strait,China, 22.7993 N, 116.7812 E | KX611426 |
| *C. mitra* | P10A1 | May 27, 2010 | Tromsø Harbour, 69.6519 N 18.9533 E, Norway | KX611427 |
