# Supplementary material for: Diversity in the Globally Distributed Diatom Genus Chaetoceros (Bacillariophyceae): Three New Species from Warm-Temperate Waters
Source: PLoS One. 2017 Jan 13;12(1):e0168887. doi: 10.1371/journal.pone.0168887 (PMC5235366; doi:10.1371/journal.pone.0168887)
Supplement: S3 Table — Evaluation of previously published records of species in the C. lorenzianus complex using EM. (DOCX) [file pone.0168887.s006.docx]

**Table S3. Evaluation of previously published records of species in the *C. lorenzianus* complex using EM**

| **Our identification** | **Previous identification** | **Reason for change or support** | **Reference** |
| --- | --- | --- | --- |
| *C. decipiens* | *C. decipiens* | Fusion of setae | Omura et al. 2012 |
| *C. decipiens* | *C. decipiens* | Fusion of setae | Berard-Therriault et al. 1999 |
| *C. decipiens* | *C. decipiens* | Fusion of setae | Lee et al. 2012 |
| *C. decipiens* | *C. decipiens* | Fusion of setae | Hoppenrath et al. 2009 |
| *C. decipiens* | *C. decipiens* | Fusion of setae | Hendey 1954 |
| *C. decipiens* | *C. decipiens* | Fusion of setae | Orlova et al. 1988 |
| *C. decipiens* | *C. decipiens* | Fusion of setae, setae in apical plan | Rines & Hargraves 1988 |
| *C. decipiens* | *C. decipiens* | Fusion of setae, oval shape and size of setae poroids | Jensen & Moestrup 1998 |
| *C. decipiens* | *C. decipiens* | Fusion of setae, narrow apertures, shape of seta poroids, lack of external rimoportual tube | Shevchenko et al. 2006 |
| *C. decipiens* | *C. decipiens* | Fusion of setae | Sunesen et al 2008 |
| *C. mitra* | *C. lorenzianus* | Small size and higher density (50-55 in 10 µm) of oval-shaped setae poroids, morphology of resting spores | Jensen & Moestrup 1998 (loc. cit. figs 160-165) |
| *C. decipiens* | *C. decipiens* | Brunel group I, fusion of setae and the oval shape of the setae poroids | Hernández-Becerril 1996 (loc. cit. Pl.20, figs 2, 3, Pl.21, figs 1, 2) |
| *C. decipiens* | *C. decipiens* | Fusion of setae and the oval shape of the setae poroids | Hernández-Becerril & Granados 1998 |
| *C. decipiens* | *C. lorenzianus* | Poroid size, reduced rimoportula process and molecular data | Kooistra et al. 2010 (loc. cit. figs 71-75) |
| *C. elegans* | *C. decipiens* | Drop-shaped setae poroids, and setae poroid density (7-13 in 10 µm) | Okuno 1956 (loc. cit. figs 7, 8) |
| *C. elegans* | *C. lorenzianus* | Large aperture, distinct basal part of setae and drop-shaped poroids on the setae | Hernández-Becerril 1996 (loc. cit. Pl.22, figs 3, 6) |
| *C. elegans* | *C. decipiens* | Seta poroid size, shape and density | Lee et al. 2014a (Loc. Cit. figs 77-80) |
| *C. laevisporus* | *C.* cf. *lorenzianus* | Poroid size and molecular data | Kooistra et al. 2010 (loc. cit. figs 77-79) |
| *C. mannaii* | *C. lorenzianus* | Heavily silicified valve, robust setae with large poroids, rimoportula with distinct external tube and short basal part of setae, hexagonal apertures | Shevchenko et al. 2006 (loc. cit. figs 84-86, 88) |
| *C. mannaii* | *C. lorenzianus* | Setae fused outside chain margin, hexagonal aperture, seta poroid size and shape | Lee et al. 2014a |
